# Nursing teleconsultation in primary health care: scoping review*

**DOI:** 10.1590/1518-8345.7212.4329

**Published:** 2024-09-02

**Authors:** Vitória Lídia Pereira Sousa, Francisco Wellington Dourado, Saiwori de Jesus Silva Bezerra dos Anjos, Andréa Carvalho Araújo Moreira

**Affiliations:** 1 Universidade Estadual do Ceará, Centro de Ciências da Saúde, Fortaleza, CE, Brazil.; 2 Universidade Estadual Vale do Acaraú, Centro de Ciências da Saúde, Sobral, CE, Brazil.

**Keywords:** Remote Consultation, Telenursing, Primary Health Care, Nursing Care, Nursing, Primary Care Nursing

## Abstract

**Objective:**

to map nurses’ skills for nursing teleconsultation in Primary Health Care.

**Method:**

this is a scoping review guided by the recommendations of the Joanna Briggs Institute Reviewer’s Manual, carried out in seven databases and repositories of theses and dissertations. The selection of studies was carried out in Rayyan by two independent, blind reviewers. Data analysis was carried out descriptively.

**Results:**

23 studies were selected, which showed that the skills necessary for nursing teleconsultation in primary care were: communication, clinical, technological and ethical. The lack of digital infrastructure was identified as one of the main barriers to the implementation of teleconsultation. The lack of access to information and communications technology and/or the internet, the severity of the clinical condition and the patient’s non-adherence to the remote consultation were also identified.

**Conclusion:**

nursing teleconsultation in primary care is an emerging way of providing health care. However, for its implementation it is necessary to train nurses in the following skills: communication, clinical, technological, ethical and those related to the infrastructure of the teleconsultation environment.

## Highlights

**(1)** Nursing teleconsultation is an emerging way of providing healthcare.

**(2)** Teleconsultation saves time and resources, achieving high levels of resolution.

**(3)** For its implementation, training is necessary to qualify nurses.

**(4)** Professional skills are required to carry out teleconsultation.

**(5)** Teleconsultation is considered a complementary offer to in-person consultation.

## Introduction

Teleconsultation can be defined as a remote consultation that includes interactions between a healthcare professional and a patient with the aim of providing diagnostic or therapeutic advice electronically^1^. 

This type of care is expanding in many countries, with the main factors involved being concern about reducing healthcare costs and current epidemiological factors, such as population aging, the increase in chronic diseases and infectious diseases^2^.

It appears that the use of teleconsultation to assist in the provision of clinical nursing care remotely has increased significantly in recent years, with great potential for application in public health emergency contexts. The context of the coronavirus disease 2019 (COVID-19) pandemic contributed to a change in the traditional care model, nurses had to give up routine face-to-face care and invest in technological solutions to carry out non-face-to-face clinical monitoring of patients^3^.

Telehealth use in the United States increased during the pandemic to about 50% of health care appointments, and the proportion should be about 20% post-pandemic, corresponding to an increase over previous estimates of 14% between 2014 and 2020^4^. The American Telemedicine Association projects that more than half of all healthcare services will be provided virtually by 2030, given that patients who have adapted to this modality of care expect to continue to receive care through it^5^.

An important measure in the Brazilian scenario, in the field of telenursing, was the Resolution of the *Conselho Federal de Enfermagem* (COFEN), which authorized the carrying out of nursing teleconsultation during a pandemic caused by the new *Severe Acute Respiratory Syndrome Coronavirus* 2 (SARS-CoV-2)^6^. Subsequently, based on the repercussions of the use of technologies to provide nursing care, the aforementioned Council decided to standardize the practice of telenursing in Brazil, through a new COFEN Resolution nº 696/2022, establishing rules for action in digital health in the scope of the SUS, as well as in supplementary and private health^7^. 

The regulation of telenursing is considered a historic milestone for the profession, as it contributes to the consolidation of this care practice outside the public emergency scenario and guarantees its applicability in an ethical and legal manner^8^. In this way, the Resolution ensures that nurses can carry out teleconsultations safely and enables them to act in various scenarios.

Within the scope of Primary Health Care (PHC), nursing teleconsultation supports the attributes of first contact, longitudinality, comprehensiveness and coordination, as it expands users’ access to health services, promoting viable care, which can be of quality, safe and effective. Furthermore, it is applicable in different lines of care^4^ ^),(^ ^9^. During the pandemic, PHC nurses demonstrated skills in providing clinical care remotely, being able to assess the general state of health, recognize signs of worsening and offer active listening via telecare, in addition to recognizing and applying protocols and clinical guidelines^9^. 

However, such technologies should not operate only as an additional service to the care network or only during pandemic periods. The benefits of telehealth tools for the system point to the importance of them being adopted more comprehensively in the *Sistema Único de Saúde* (SUS) and, in particular, in an integrated way with PHC^10^. In view of this, it is necessary to identify how nursing teleconsultation has been carried out in PHC, in order to identify the main skills for carrying it out, as well as the advantages and limitations of this type of care, aiming to facilitate its effective implementation. 

In view of the above, the present study aims to map nurses’ skills for nursing teleconsultation in Primary Health Care. It is expected that this study will provide insights to expand knowledge and understanding about nursing teleconsultation. 

## Method

### Study design

This is a scoping review guided by the recommendations of the Joanna Briggs Institute Reviewer’s Manual (JBI)^11^. The research protocol was registered in the Open Science Framework (OSF) (https://osf.io/tpvbg/), and the Systematic Reviews and Meta-Analyses for Scoping Reviews (PRISMA-ScR) extension was used to report the results of the scope analysis^12^.

To carry out the review, the following steps were considered: Phase 1 - Eligibility criteria; Phase 2 - Sources of information and literature search; Phase 3 - Selection of evidence sources; Phase 4 - Data extraction; and Phase 5: Data analysis and presentation.

#### Phase 1 - Eligibility criteria

The eligibility criteria were based mainly on the components of the PCC strategy, which served as the basis for constructing the guiding question, namely: Population (P): refers to nurses; Concept (C): nursing teleconsultation; Context (C): Primary Health Care. Thus, the following question was defined: What are nurses’ skills for nursing teleconsultation in Primary Health Care? 

The inclusion criteria were: studies published in full, available electronically, without time or language restrictions, whose research presents nurses’ skills for nursing teleconsultation in Primary Health Care. Editorials, letters to the editor, videos, websites, news, preprints and abstracts were deleted.

#### Phase 2 - Sources of information and literature search

To outline the search strategy, the following steps were used: extraction, conversion, combination, construction and use (Figure 1)^13^. The words were chosen based on the terms present in the PCC strategy, through a search in health thesauruses, such as Medical Subject Headings (MeSH) and Health Sciences Descriptors (DeCS) and Embase Subject Headings (Emtree).

**Figure 1:** Search strategy. Fortaleza, CE, Brazil, 2023

Objective/Problem	What are nurses’ skills for nursing teleconsultation in Primary Health Care?		
	P*	C^†^	C^‡^
**Extraction**	Nurse	Nursing teleconsultation	Primary Health Care
**Conversion**	*Nursing*	*Remote consultation*	*Primary Health Care*
**Combination**	*Nursing; nurses; nurse; nursing care; Nursing, Primary; Primary Nursing Care; Care, Primary Nursing; Nursing Care, Primary*	*Remote consultation; Consultation, Remote; Teleconsultation; Teleconsultations; Asynchronous teleconsultation*	*Primary Health Care; Care Primary Health; Health Care Primary; Primary Healthcare; Primary Care;*
**Construction**	(“Nursing” OR “nurses” AND “nurse” OR “nursing care” OR “Nursing, Primary” OR “Primary Nursing Care” OR “Care, Primary Nursing” OR “Nursing Care, Primary”)	(“Remote consultation” OR “Consultation, Remote” OR “Teleconsultation” OR “Teleconsultations” OR “Asynchronous teleconsultation”)	(“Primary Health Care” OR “Care Primary Health” OR “Health Care Primary” OR “Primary Healthcare” OR “Primary Care”)
**Use**	(“Nursing” OR “nurses” AND “nurse” OR “nursing care” OR “Nursing, Primary” OR “Primary Nursing Care” OR “Care, Primary Nursing” OR “Nursing Care, Primary”) AND (“Remote consultation” OR “Consultation, Remote” OR “Teleconsultation” OR “Teleconsultations” OR “Asynchronous teleconsultation”) AND (“Primary Health Care” OR “Care Primary Health” OR “Health Care Primary” OR “Primary Healthcare” OR “Primary Care”)		

*P = Population; ^†^C = Concept; ^‡^C = Context

To make the search as sensitive as possible, the primary descriptors “Remote consultation”, “Nursing Care” and “Primary Health Care” were used, in Portuguese and English. To expand the search and better target the findings, synonymous terms and the Boolean operators OR and AND were used, adapting the terms according to the specificity of each base.

The research strategy and the entire process of preparing this work adopted the systematic scoping review methodology proposed by the Joanna Briggs Institute^11^. Therefore, a three-step search strategy was used: I) Limited initial search in the MEDLINE/PubMed databases, followed by an analysis of the words in the titles, abstracts and indexing terms used to describe the article; II) Second search using all keywords and indexing terms identified in the included databases; and III) The references of all articles and reports found in the search were analyzed to identify additional studies.

The databases used were: Medical Literature Analysis and Retrieval System Online (MEDLINE), Cochrane Library, Web of Science, Latin American and Caribbean Health Sciences Literature (LILACS), *Excerpta Medica Database* (EMBASE), SciVerse Scopus (SCOPUS) and Cumulative Index to Nursing and Allied Health Literature (CINAHL). In addition, gray literature was included. 

The gray literature evidence was investigated in the Catalog of Theses and Dissertations of the *Coordenação de Aperfeiçoamento de Pessoal de Nível Superior* (CAPES), Google Scholar, searches on websites of official bodies, manuals from international and national institutions, guidelines and books. 

The search and the selection of studies occurred from February to April 2023.

#### Phase 3 - Selection of evidence sources

After searching information sources, the studies were exported to the Rayyan application (Rayyan Systems Inc, Cambridge, MA, USA) to remove duplicate documents and select studies. When selecting studies, two independent evaluators analyzed the titles and abstracts, following established criteria for inclusion. The full texts of potentially relevant studies underwent a thorough analysis by the evaluators, maintaining the same inclusion criteria. In cases of doubts or disagreements, a third reviewer specialized in the area of the object of study issued their opinion.

#### Phase 4 - Data extraction

To extract the data, a standardized instrument previously prepared by the authors was used, containing information about the characterization of the studies (title of the article; author(s); year of publication; origin/country of study; language; database; journal; reference; objective or research question; methodology/methods/type of study; population and sample; main results). 

#### Phase 5 - Data analysis and presentation

To compile and present the results, a chart was created with the main characteristics of the studies, as well as a qualitative thematic analysis was carried out to provide an overview of the literature on nurses’ skills for nursing teleconsultation in PHC. 

### Ethical aspects

As this was research using secondary data, in the public domain and available in the literature, there was no need for ethical assessment. However, it should be noted that copyright was respected with correct citation and referencing of studies.

## Results

**Figure 2:**
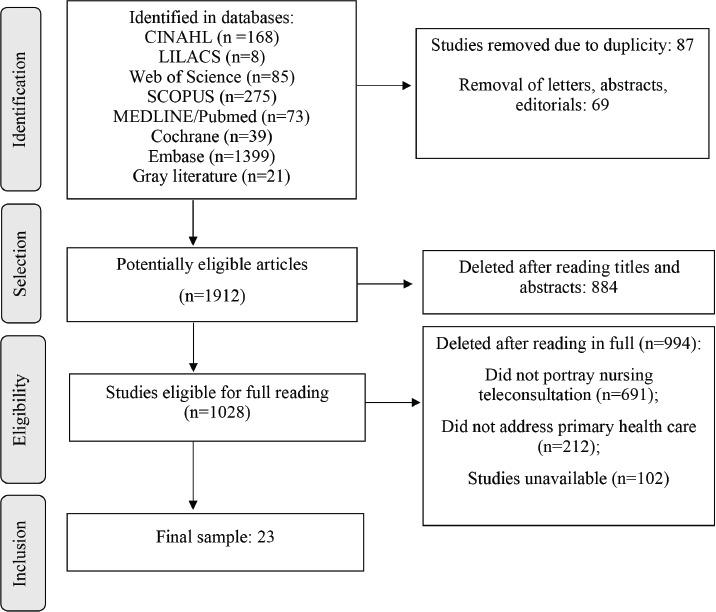
Database search flowchart. Fortaleza, CE, Brazil, 2023

Firstly, 2068 publications were identified. After applying the inclusion criteria and reading the titles and abstracts, 1912 potentially eligible studies were selected, and subsequently 1028 to be analyzed in full. Of these, 23 constituted the study sample. To describe the searches and select studies, the Preferred Reporting Items for Systematic Review and Meta-Analyses (PRISMA) was used (Figure 2).

The studies identified were mostly from countries on the European continent (n=13), from the United Kingdom (n=5), Spain (n=3), Sweden (n=2), the Netherlands (n=2) and Scotland (n=1), and from countries in America (n=8), specifically Brazil (n=5), Canada (n=2) and the United States (n=1). Furthermore, productions from the Asian continent (n=2) were identified, specifically from Singapore (n=1) and India (n=1). 

Regarding the design of the studies, most had qualitative approaches of the following types: descriptive research (n= 09); experience report (n=01); case study (n=01) and theoretical-reflective study (n= 01). The rest of the productions had a quantitative approach, and consisted of: randomized clinical trial (n=2); cohort (n=3); and transversal (n=2). In addition, three guidelines were identified. Dissertations and theses were not included in the final sample, as they did not answer the research question.

Concerning the period of publication, these are recent articles. There was a predominance of studies published in the last three years (2020-2022), which totaled 17 productions. In the period from 2012 to 2016, 4 studies were identified. The other two articles dated 2007 and 2009. Such results reinforce the fact that this is a topic of increasing interest in recent years. The summary of the identified articles is described in Figure 3.

**Figure 3:** Characterization of articles identified in the review. Fortaleza, CE, Brazil, 2023

Number	Title	Type of study	Year	Country	Sample
A1^14^	Nurse practitioner-based diabetes care management: Impact of telehealth or telephone intervention on glycemic control.	Retrospective cohort study	2007	United States	259
A2^15^	Telephone consulting in primary care: a triangulated qualitative study of patients and providers.	Descriptive study with a qualitative approach	2009	Scotland	91
A3^16^	Nurse telephone triage in Dutch out-of-hours primary care: the relation between history taking and urgency estimation.	Cohort study	2012	Netherlands	304
A4^17^	Question design in nurse-led and GP-led telephone triage for same-day appointment requests: a comparative investigation.	Randomized controlled study	2014	United Kingdom	51
A5^18^	Competencies required for nursing telehealth activities: A Delphi-study.	Delphi Method	2016	Netherlands	51
A6^19^	Physical Examinations via Video for Patients With Heart Failure: Qualitative Study Using Conversation Analysis.	Descriptive study with a qualitative approach	2020	United Kingdom	07
A7^20^	Registered nurses´ views on telephone nursing for patients with respiratory tract infections in primary healthcare - a qualitative interview study.	Descriptive study with a qualitative approach	2020	Sweden	12
A8^21^	Consensus on Criteria for Good Practices in Video Consultation: A Delphi Study.	Cross-sectional study	2020	Spain	16
A9^22^	Remote Consultations Guidance Under COVID-19* Restrictions.	Guidelines	2020	United Kingdom	-
A10^23^	Telenursing Practice Guidelines.	Guidelines	2020	India	-
A11^24^	*Guia de orientação para teleconsulta de enfermagem*.	Guidelines	2020	Brazil	-
A12^25^	Implementation of remote consulting in UK primary care following the COVID-19* pandemic: a mixed-methods longitudinal study.	Cohort study	2021	United Kingdom	41
A13^26^	Video-consultation in primary health care: an implementation experience.	Descriptive study with a quantitative approach	2021	Spain	76
A14^27^	Implementation of COVID-19* telemonitoring: repercussions in Nursing academic training.	Descriptive study with a qualitative approach	2021	Brazil	17
A15^28^	Telehealth in Primary Healthcare: A Portrait of its Rapid Implementation during the COVID-19* Pandemic.	Cross-sectional study	2021	Canada	603
A16^29^	Nurse-led telephone intervention for lifestyle changes on glycaemic control in people with prediabetes: Study protocol for a randomized controlled trial.	Randomized controlled study	2021	Spain	428
A17^30^	Telemonitoring programme on COVID-19* for a low-income community in Brazil: case study.	Descriptive study with a qualitative approach	2021	Brazil	1076
A18^31^	Remote Consulting in Primary Health Care in Low- and Middle-Income Countries: Feasibility Study of an Online Training Program to Support Care Delivery During the COVID-19* Pandemic.	Descriptive study with a qualitative approach	2022	United Kingdom	75
A19^32^	Right siting of complex acute wound management---preliminary study of teleconsultation wound services between acute and primary care in Singapore.	Descriptive study with a quantitative approach	2022	Singapore	18
A20^33^	Electronic Consultation by Advanced Practice Nurses to Improve Access to Specialist Care for Older Adults.	Multiple case study	2022	Canada	06
A21^34^	*Relato de experiência das contribuições da telessaúde em comunidades ribeirinhas do Amazonas na pandemia*.	Experience report	2022	Brazil	-
A22^35^	Saving lives by asking questions: nurses’ experiences of suicide risk assessment in telephone counselling in primary health care.	Descriptive study with a qualitative approach	2022	Sweden	15
A23^36^	Teleconsultation as an advanced practice nursing during the COVID-19* pandemic based on Roy and Chick-Meleis.	Theoretical-reflective study	2022	Brazil	-

*COVID-19 = *Coronavirus disease 2019*

Figure 4 presents the objectives of the studies and the main results regarding the characterization of nursing teleconsultation in Primary Health Care.

**Figure 4:** Descriptive summary of the studies included in the scoping review. Fortaleza, CE, Brazil, 2023

Number	Objective	Main results/Conclusion
A1^14^	Compare the impact of nurse-led diabetes *mellitus* care management programs using telehealth or telephone intervention.	Teleconsultations were used to initiate and adjust medications, order laboratory tests, review and discuss laboratory results, and encourage lifestyle changes. Teleconsultation can provide better adherence to treatment plans.
A2^15^	Understand patient and healthcare team perspectives on how telephone consultations differ from face-to-face consultations in terms of content, quality and safety.	Although telephone consultation generally provides better access, the quality of the consultation can potentially be compromised, particularly due to the lack of formal and informal examinations. Telephone consultation was considered more appropriate for monitoring chronic diseases.
A3^16^	Examine the relation between the comprehensiveness of the anamnesis and the adequacy of the urgency estimate.	Two types of questions can be distinguished: discriminative and general. Pattern recognition is more important for nurses to identify urgent health problems than asking all the crucial questions during history taking.
A4^17^	Compare doctors’ and nurses’ communication with patients in telephone triage consultations in primary care.	Although nurses ask three times as many questions as doctors, the similarity in the length of triage calls is explained by the content and form of the questions used. Questions designed for yes or no should be avoided, so that they do not seem like a checklist and do not make interaction between patient and nurse impossible.
A5^18^	Identify what skills are needed to provide telehealth.	Communication skills, coaching skills, ability to combine clinical experience with telehealth, clinical knowledge, ethical awareness and supportive attitude were seen as the most important competencies for nurses providing telehealth.
A6^19^	Explore the opportunities and challenges of remote physical examination of patients with heart failure using video-mediated communication technology.	Remote physical examination in patients with heart failure is restricted to evaluating certain conditions such as fluid retention, blood pressure rate and pulse rhythm, and oxygen saturation. Therefore, video exams are possible in the context of heart failure services.
A7^20^	Describe registered nurses’ views of telephone nursing work with callers to primary health care centers regarding respiratory tract infections.	During teleconsultation, nurses must establish good communication with the patient; differentiate harmless from serious problems; deal with user expectations; and use work tools.
A8^21^	Develop consensual criteria for the management of video consultations that contribute to effective and quality healthcare provided by healthcare professionals.	During the teleconsultation, basic elements must be taken into account, such as technical resources, the relationship established between health professionals and patients, and the environment during their interaction.
A9^22^	Support nursing staff to see and/or treat patients via telephone or video or through other remote consultation processes.	The decision to offer a remote consultation versus an in-person consultation should be based on the initial screening process. It is necessary to assess the severity of physical and psychological symptoms.
A10^23^	Provide general guidance to registered nurses on using telehealth technology.	The use of cameras allows for a simple and inexpensive teleconsultation experience. Communication during the teleconsultation must be effective. It is essential to record the teleconsultation, describing the stages of the nursing process.
A11^24^	Support professional nurses in the clinical practice of Nursing Teleconsultation, considering COFEN* Resolution number 0634/2020.	The Consent Form aims to protect nurses and patients, so it should always be requested. It is necessary to have a private and bright room for the professional; cell phone or computer with camera; and internet connection.
A12^25^	Investigate the implementation of remote consultation and explore the impact in the early months of the COVID-19^†^pandemic.	Nurses found that telephone consultation worked well for reviews of chronic conditions, prioritizing poorly controlled patients and seeing in-person patients only for physical aspects.
A13^26^	Report the implementation of video consultation in PHC^‡^.	To carry out a video consultation it is necessary to have specific technology: a program to generate virtual meetings, a computer, tablet and/or cell phone with integrated camera and microphone.
A14^27^	Report the repercussions of implementing monitoring of suspected and confirmed cases of COVID-19^†^ on academic nursing education.	Nurses need to be sensitive to what is said between the lines, as assessment is the basis for knowing whether the patient needs direct care or is referred further in the care system. It is necessary to carry out a good assessment of the user’s health status, through data collection and observation.
A15^28^	Document the adoption of telehealth by various types of primary health care providers during the COVID-19^†^ pandemic and identify the advantages and disadvantages of telehealth.	Many PHC^‡^ nurses were concerned about the lack of eye contact during telephone consultations. As an alternative to providing visual contact, it was suggested to send photos. The physical examination remains an essential element in evaluating more complex problems.
A16^29^	Evaluate the effectiveness of a nurse-led telephone intervention for lifestyle change.	Evidence indicates that nursing teleconsultation in PHC^‡^ has been satisfactory, mainly for the evaluation of patients with chronic non-communicable diseases, and patients with greater difficulty controlling the disease should be prioritized.
A17^30^	Report the process of creating and implementing a monitoring program for patients with a confirmed or suspected diagnosis of COVID-19^†^ through telemonitoring.	The teleconsultation program was implemented through three phases: planning, implementation and monitoring. It was identified that the professional needs to have clinical skills to recognize signs and symptoms of severity.
A18^31^	Determine whether “remote consultation in primary health care” training is acceptable and feasible for health professionals in rural Tanzania to support health care delivery during the pandemic.	Professionals expressed that they learned skills necessary to consult remotely within the health system. And there was greater understanding of topics such as ethics in remote consulting.
A19^32^	Provide preliminary evidence on the feasibility of the inaugural use of teleconsultation between hospitals and primary care services for acute wound management in Singapore.	The management of complex acute wounds is challenging, time-consuming and expensive. The wound teleconsultation service is viable and acceptable, and has been shown to be effective for wound healing, in addition to being potentially cost-effective.
A20^33^	Explore how nurses’ use of the eConsult service can improve access to specialized care for older adults in a variety of settings.	In the context of an aging population, eConsult can empower the nurse to serve as an effective healthcare human resource who can address the unique access issues faced by older adults in primary care.
A21^34^	Report the creation and implementation of telehealth activities developed by the *Programa Saúde na Floresta* in communities in conservation areas, in the state of Amazonas, during the COVID-19^†^ pandemic.	For nursing, the most requested specialty was in the area of obstetrics, followed by pediatrics. Such actions reduced the circulation of these populations in large urban centers, which promoted extra immunity due to the social isolation facilitated by the use of telehealth.
A22^35^	Explore nurses’ experiences in assessing suicide risk in telephone counseling in primary health care.	Teleconsultations do not allow physical examinations, increasing the risk of errors, therefore, the nurse must use experience, knowledge and critical thinking to identify warning signs.
A23^36^	Carry out a reflective analysis based on the theoretical contributions of the Adaptation model, by Roy, and the Transition model, by Chick-Meleis, and also on the contribution of teleconsultation as an ANP^§^ in the care of elderly patients and those with chronic illnesses.	Teleconsultation is an advanced nursing practice that requires nurses to fully develop their clinical reasoning to implement a well-designed nursing process, which must be anchored in a consistent theoretical framework.

*COFEN = *Conselho Federal de Enfermagem*; ^†^COVID-19 = Coronavirus disease 2019; ^‡^PHC = Primary Health Care; ^§^ANP = Advanced Nursing Practice

Given the results, it was possible to identify different types of skills needed for nursing teleconsultation, as well as evidence on the potentialities and the barriers of teleconsultation in PHC and operational aspects of the nursing process in teleconsultation. From a qualitative point of view, for a better understanding and organization of the results, they were subdivided into three thematic categories: Skills for nursing teleconsultation in PHC, Potentialities and barriers of nursing teleconsultation in PHC and Nursing process in teleconsultation. Figure 5 presents the thematic categories.

**Figure 5:** Scoping review thematic categories. Fortaleza, CE, Brazil, 2023

Thematic categories
Skills for nursing teleconsultation in PHC
Communication^14^ ^),(^ ^16^ ^)-(^ ^25^ ^),(^ ^27^ ^)-(^ ^30^ ^),(^ ^35^, clinical^15^ ^)-(^ ^25^ ^),(^ ^27^ ^)-(^ ^28^ ^),(^ ^30^ ^),(^ ^32^ ^),(^ ^36^, technological^15^ ^),(^ ^19^ ^),(^ ^21^ ^)-(^ ^24^ ^),(^ ^26^ ^)-(^ ^28^ ^),(^ ^31^ ^),(^ ^34^ ^),(^ ^36^, ethical^15^ ^),(^ ^18^ ^),(^ ^21^ ^)-(^ ^24^ ^),(^ ^27^ ^)-(^ ^28^ ^),(^ ^31^ ^),(^ ^35^ skills, and those related to infrastructure^18^ ^)-(^ ^19^ ^),(^ ^21^ ^)-(^ ^26^ ^),(^ ^31^ ^),(^ ^33^.
**Potentialities and barriers of nursing teleconsultation in PHC**
Potentialities - Ease of access^14^ ^),(^ ^16^ ^)-(^ ^25^ ^),(^ ^27^ ^)-(^ ^36^, monitoring patients with COVID-19^22^ ^)-(^ ^25^ ^),(^ ^27^ ^)-(^ ^28^ ^),(^ ^30^, diabetes *mellitus* ^14^ ^),(^ ^28^ ^)-(^ ^29^, arterial hypertension^28^, respiratory tract infection^20^, sexually transmitted infections^31^, heart failure^19^, wound assessment^25^ ^),(^ ^32^, mental health^35^, health of elderly people^33^, child health^34^.
Barriers - Lack of access to ICT* and/or the internet^15^ ^),(^ ^21^ ^)-(^ ^24^ ^),(^ ^26^ ^),(^ ^33^ ^),(^ ^36^, severity of the clinical condition^15^ ^)-(^ ^17^ ^),(^ ^20^ ^)-(^ ^24^ ^),(^ ^27^ ^)-(^ ^28^ ^),(^ ^30^ ^),(^ ^33^ ^),(^ ^35^, patient’s non-adherence^22^ ^)-(^ ^24^ ^),(^ ^33^ ^),(^ ^36^.
**Nursing process in teleconsultation**
Assessment^16^ ^)-(^ ^17^ ^),(^ ^20^ ^)-(^ ^25^ ^),(^ ^35^, physical exam^14^ ^),(^ ^19^ ^),(^ ^24^ ^)-(^ ^25^, diagnostics^19^ ^)-(^ ^20^ ^),(^ ^24^ ^),(^ ^36^, planning^24^, prescription^23^ ^)-(^ ^25^ ^),(^ ^28^ ^),(^ ^31^ ^),(^ ^36^, evolution^22^ ^)-(^ ^24^ ^),(^ ^27^ ^),(^ ^31^ ^),(^ ^36^.

*ICT = Information and Communications Technology

## Discussion

The findings are presented and discussed in three thematic categories: Skills for nursing teleconsultation in PHC, Potentialities and barriers of nursing teleconsultation in PHC and Nursing process in teleconsultation.

### Skills for nursing teleconsultation in PHC

To guarantee the quality of health care through nursing teleconsultation, studies indicate the need for a set of skills, among which the following stand out: communication, clinical, technological, ethical and those related to the infrastructure of the teleconsultation environment^18^ ^),(^ ^21^ ^)-(^ ^24^ ^),(^ ^36^. 

Communication was highlighted as the main skill for carrying out teleconsultations. The quality of the interaction between professional and patient during teleconsultation is fundamental to aspects of safety, effectiveness, patient experience and, potentially, health outcomes^17^ ^),(^ ^20^ ^),(^ ^27^. Evidence shows that communication needs to be sufficient to identify the patient’s symptoms and assess their health needs, making this an even more difficult task when it comes to an audio call, as the lack of visual information and the need to rely only on the verbal information increases nurses’ concern^20^ ^),(^ ^27^ ^)-(^ ^28^ ^),(^ ^35^. 

Another skill identified as necessary is clinical, as teleconsultation requires nurses to have clinical reasoning to implement a well-designed nursing process, remotely^20^ ^),(^ ^27^ ^),(^ ^36^. Clinical skill refers to the ability to interpret human responses accurately to select appropriate interventions and evaluate the outcome achieved, and involves the professional’s clinical knowledge and previous experiences^18^ ^),(^ ^21^. 

The importance of clinical skill is highlighted for the process of interpretation and grouping of collected data, which culminates in decision-making about nursing diagnoses and that constitutes the basis for the selection of actions or interventions with which the objective is to achieve the expected results^36^.

It is necessary for nurses to have technological abilities to carry out teleconsultations, and must know basic computer skills, know how to handle technological devices (cell phone, notebook, tablet), be able to check the functionality of communication equipment and train patients to use Information and Communications Technology (ICT)^21^ ^),(^ ^28^. 

Having skills and ethically correct attitudes such as honesty, confidentiality, and personal and professional integrity are essential during nursing teleconsultation. The nurse must ensure the patient’s privacy under any circumstances and guarantee the confidentiality of information obtained through the therapeutic relationship^18^ ^),(^ ^26^ ^),(^ ^31^.

The nurse must guarantee the privacy and protection of information, with disrespect being a violation of the dignity of patients^18^ ^),(^ ^26^ ^),(^ ^31^. Teleconsultation implies greater risks of data breach than in-person service, in this sense, it is essential to comply with current regulations, with emphasis on the *Lei Geral de Proteção de Dados* (LGPD), nº 13.709/2018, which has been in force since 2020 in Brazil and has been driving changes in care processes and technologies^37^. 

Preparing the environment where the nursing teleconsultation will take place is essential to guarantee the quality of the consultation, and the nurse must have this skill. If properly prepared, it is possible to reduce social distance and establish a greater bond with the patient, considering that barriers typical of a healthcare environment, such as table and examination table, do not exist in the scenario of a teleconsultation, increasing the quality of communication and trust between healthcare professionals and patients^21^ ^)-(^ ^22^ ^),(^ ^24^.

It is essential that, before starting the teleconsultation, the quality of the connection and the correct functioning of the audio and/or video are checked, so that such factors do not compromise the consultation^22^ ^),(^ ^24^ ^),(^ ^31^. The nurse must be prepared in cases of internet connection failure and maintain a proactive attitude when resolving technical problems^21^ ^),(^ ^31^.

### Potentialities and barriers of nursing teleconsultation in PHC

Scientific evidence points to the potential of nursing teleconsultation in PHC, mainly because it facilitates access in remote areas. However, for it to be carried out safely and effectively, it is necessary to identify which patients can be assisted by this type of care. The studies address nursing teleconsultation in different lines of care in PHC, such as monitoring patients with COVID-19, diabetes *mellitus*, arterial hypertension, respiratory tract infections, sexually transmitted infections, heart failure, wound assessment, mental health, health of elderly people, and child health^14^ ^)-(^ ^15^ ^),(^ ^19^ ^)-(^ ^20^ ^),(^ ^22^ ^)-(^ ^25^ ^),(^ ^27^ ^)-(^ ^30^ ^),(^ ^32^ ^)-(^ ^36^. 

A study carried out in the United States showed that nursing teleconsultation, when used to provide individualized diabetes care management, has similar effects on glycemic control when compared to in-person consultation^14^. Another study carried out in the United Kingdom showed that telephone nursing teleconsultation worked well for reviews of chronic conditions, with face-to-face care only being necessary to assess physical aspects^25^.

Given the potential and dissemination of teleconsultation in PHC, its applicability to patients with diverse complaints and demands can be observed. Despite the different approaches, it was identified in common among the studies that nursing teleconsultation in PHC has been satisfactory, providing a better clinical condition for the patient. 

Factors such as lack of access to ICT and/or the internet, severity of the clinical condition, or the patient’s non-adherence to the remote consultation prevent it from being carried out^22^ ^)-(^ ^24^ ^),(^ ^33^. The lack of access to ICT is prevalent among the population, being one of the barriers to the implementation of teleconsultation, which is why it must be checked in advance whether the patient has sufficient and appropriate technological resources to carry out the teleconsultation^15^ ^),(^ ^21^ ^)-(^ ^24^ ^),(^ ^26^ ^),(^ ^33^ ^),(^ ^36^. 

Nurses are prohibited from carrying out teleconsultations to deal with urgent or emergency situations^8^. When warning signs are identified, care should not be continued, but the search for an emergency service should be advised^23^ ^)-(^ ^24^ ^),(^ ^33^. It is the nurse’s responsibility to know the *Rede de Atenção à Saúde* (RAS) available in the territory of the patient being treated, so that, in the event of any need for referral, the appropriate service can be guided for each situation^8^.

As it is a type of care considered recent, it is common for patients to choose not to join the teleconsultation, therefore, the patient’s consent form is considered a requirement for carrying out the teleconsultation^22^ ^)-(^ ^24^ ^),(^ ^36^. According to COFEN Resolution nº 696/2022, which provides for the role of Nursing in Digital Health, standardizing Telenursing, the consent of the user/patient involved or their legal guardian is essential, and that it is carried out by their free decision, being subject to withdrawal at any time and, consequently, withdrawal of consent^6^.

### Nursing process in teleconsultation

The teleconsultation must follow the same execution method used in the face-to-face Nursing consultation, considering the Nursing Process, including the steps: nursing assessment, nursing diagnosis, nursing planning, implementation and nursing evolution^7^ ^),(^ ^38^.

During the nursing assessment, it is recommended that questions for data collection be asked through open questions and collected, preferably, from the patient, with their consent being requested if necessary to include the family member/caregiver. It is important to avoid questions designed for yes or no, so that they do not seem like a checklist and do not make interaction between patient and nurse impossible. Furthermore, the use of questions that lead the patient to a specific answer should be avoided, in order to enable better clinical assessment^17^ ^),(^ ^21^ ^)-(^ ^22^ ^),(^ ^35^.

The fact that patient and professional do not share a physical environment makes direct physical examination impossible and limits assessment^19^. Despite this, studies indicate that through video teleconsultation it is possible to inspect the patient and evaluate some aspects: identifying changes in the skin, presence of edema, general appearance/appearance, respiratory rate, eye irritation, muscle strength and mobility, and presence of heavy bleeding^14^ ^),(^ ^19^ ^),(^ ^24^.

Regarding the type of call to carry out nursing teleconsultation, some of the studies indicated that the choice must be made according to the patient’s clinical condition. It is recommended that some criteria be considered, for example, patients with less clinically complex cases can generally be contacted by telephone^15^ ^),(^ ^26^. In turn, video calling is more suitable for patients with greater complexity and the presence of comorbidities, as well as for patients with hearing impairment^23^ ^),(^ ^26^.

After carrying out the nursing assessment, it is necessary to identify nursing diagnoses and define a care plan for the patient. When trained, the nurse is able to carry out a quality nursing teleconsultation, identifying accurate diagnoses and nursing problems, using wisely intentional questions, and proposing relevant interventions to achieve the expected results^36^.

Nursing evolution comprises the evaluation of the results achieved in nursing and health of the person, family, community and special groups. This step allows the analysis and review of the entire Nursing Process^38^. Studies show that nursing evolution must be carried out in detail and clearly, describing the stages of the nursing process^22^ ^)-(^ ^24^ ^),(^ ^27^ ^),(^ ^36^. 

The process of reflective monitoring of teleconsultations is recommended to analyze its conduct, aiming to improve future consultations, and it is important to identify the main flaws that may have compromised the quality of the consultation, from the analysis of technological resources as well as aspects related to the conduct of the professional in the case^21^.

It was observed that few studies address the nursing process during teleconsultation, and that, when approached, is done in a disassociated way, that is, mentioning only some of the steps. Given this, it is necessary that new studies be developed focusing on the systematization of nursing care using new technologies. It is also essential that professionals are trained to apply the systematization of nursing care remotely.

Teleconsultation is an Advanced Nursing Practice (ANP) that demands sufficient involvement from the nursing professional to get to know the patient as an individual, through the application of an instrument developed and based on a theoretical model that uses standardized taxonomies and is suitable for conducting the teleconsultation^36^.

Therefore, for the teleconsultation experience to become, in fact, a reality in PHC and SUS, a massive investment is necessary, both in guaranteeing internet access in health units and in obtaining computers and telephones, which are essential for the establishment of satisfactory communication. 

Despite its potential for use, most of the time teleconsultation is considered an additional, supplementary offer and is not used by health institutions to provide assistance. Therefore, encouraging the inclusion of its use in clinical practice on a routine basis, especially in PHC, and in the education of future health professionals, is essential to achieve adequate use.

This review highlighted scientific evidence from several countries whose PHC is organized in different ways, according to economic, social and political aspects and based on the local understanding of the health-disease process. In the European context, the concept of primary care can be understood as a level of care between informal care and hospital care. On the other hand, in some American countries, PHC refers to a specific set of health service activities aimed at the poor population. In Asian countries, the reformulation of PHC has been happening more recently, aiming for an accessible and equitable system. 

The contributions of this study to the area of Nursing and health are based on the impact of nursing teleconsultation in recent years, mainly due to the pandemic. Studies like this are incentives to reflect on the new care scenarios that nurses must act in, in addition to instigating the implementation of teleconsultation in PHC.

As a limitation, we can mention the analysis and synthesis of data carried out only in a descriptive way. Combining data from different types of studies is a challenging process that can lead to bias in the preparation of review results. Despite the limitations, this review highlights the methodological rigor required by the JBI and the mapping of evidence on nursing teleconsultation. 

## Conclusion

Nursing teleconsultation in primary care is an emerging way of providing health care. When technical and organizational difficulties are resolved, time and resources can be saved for patients and professionals, achieving satisfactory levels of problem solving, without the need to go to Basic Health Units. However, for its implementation it is necessary to train nurses in the following skills: communication, clinical, technological, ethical and those related to the infrastructure of the teleconsultation environment. 

The need for professional skills to deal with nursing teleconsultation was identified, which demands a process of continuing education that permeates the spaces of academic training, all the way to the professional practice of nurses. 
