# Supplementary material for: Nursing teleconsultation in primary health care: scoping review
Source: Rev Lat Am Enfermagem. 2024 Sep 2;32:e4329. doi: 10.1590/1518-8345.7212.4329 (PMC11386547; doi:10.1590/1518-8345.7212.4329)
Supplement: Translation

# Translation: **Teleconsulta de enfermagem na atenção primária à saúde: *scoping review*** *

**DOI:** 10.1590/1518-8345.7212.4330

**Authors:** Vitória Lídia Pereira Sousa, Francisco Wellington Dourado, Saiwori de Jesus Silva Bezerra dos Anjos, Andréa Carvalho Araújo Moreira

**Affiliations:** 1 Universidade Estadual do Ceará, Centro de Ciências da Saúde, Fortaleza, CE, Brasil.; 2 Universidade Estadual Vale do Acaraú, Centro de Ciências da Saúde, Sobral, CE, Brasil.

**Keywords:** Consulta Remota, Telenfermagem, Atenção Primária à Saúde, Cuidados de Enfermagem, Enfermagem, Enfermagem de Atenção Primária

## Abstract

**Objetivo:**

mapear as habilidades dos enfermeiros para a teleconsulta de enfermagem na Atenção Primária à Saúde.

**Método:**

trata-se de uma revisão de escopo guiada pelas recomendações do *Joanna Briggs Institute Reviewer’s Manual*, realizada em sete bases de dados e repositórios de teses e dissertações. A seleção dos estudos foi realizada no *Rayyan* por dois revisores independentes e às cegas. A análise dos dados foi realizada de forma descritiva.

**Resultados:**

selecionou-se 23 estudos, os quais mostraram que as habilidades necessárias para a teleconsulta de enfermagem na atenção primária foram: comunicação, clínica, tecnológica e ética. A falta de infraestrutura digital foi apontada como uma das principais barreiras para a implementação da teleconsulta. A falta de acesso a tecnologias da informação e comunicação e/ou à internet, a gravidade do quadro clínico e a não adesão do paciente à consulta remota também foram identificadas.

**Conclusão:**

a teleconsulta de enfermagem na atenção primária é uma forma emergente de prestar assistência à saúde. No entanto, para sua implementação é necessária a capacitação dos enfermeiros quanto às seguintes habilidades: comunicação, clínica, tecnológica, ética e aquelas relacionadas à infraestrutura do ambiente da teleconsulta.

## Destaques

**(1)** A teleconsulta de enfermagem é uma forma emergente de prestar assistência à saúde.

**(2)** A teleconsulta economiza tempo e recursos, alcançando altos níveis de resolução.

**(3)** Para sua implementação, é necessário treinamento para capacitar os enfermeiros.

**(4)** São necessárias competências profissionais para a realização da teleconsulta.

**(5)** A teleconsulta é considerada uma oferta complementar à consulta presencial.

## Introdução

A teleconsulta pode ser definida como uma consulta remota que contempla interações entre um profissional de saúde e um paciente com o objetivo de fornecer aconselhamento diagnóstico ou terapêutico por meio eletrônico^1^. 

Esta modalidade assistencial encontra-se em expansão em muitos países, tendo como principais fatores intervenientes a preocupação com a redução dos custos com cuidados de saúde e fatores epidemiológicos atuais, como o envelhecimento populacional, o aumento de doenças crônicas e os agravos infectocontagiosos^2^.

Verifica-se que a utilização da teleconsulta para auxiliar na prestação de cuidados clínicos de enfermagem à distância tem aumentado significativamente nos últimos anos, com grande potencial de aplicação em contextos de emergência de saúde pública. O contexto de pandemia da *coronavirus disease 2019* (COVID-19) contribuiu para uma mudança no modelo tradicional de atendimento, os enfermeiros tiveram de renunciar o rotineiro cuidado presencial e investir em soluções tecnológicas para realizar o acompanhamento clínico não presencial dos pacientes^3^.

O uso da telessaúde nos Estados Unidos aumentou aproximadamente 50% dos atendimentos de saúde durante a pandemia, e a proporção deve chegar próximo de 20% após a pandemia, o que corresponde a um aumento em relação às estimativas anteriores de 14% entre 2014 e 2020^4^. A *American Telemedicine Association* projeta que mais da metade de todos os serviços de saúde serão prestados virtualmente até 2030, tendo em vista que os pacientes que se adaptaram a essa modalidade de cuidado esperam continuar sendo atendidos por meio dela^5^.

Uma medida importante no cenário brasileiro, no campo da telenfermagem, foi a Resolução do Conselho Federal de Enfermagem (COFEN), que autorizou a realização da teleconsulta de enfermagem durante pandemia desencadeada pelo novo *Severe Acute Respiratory Syndrome Coronavirus* 2 (SARS-CoV-2)^6^. Posteriormente, baseado na repercussão do uso das tecnologias para a realização da assistência de enfermagem, o referido Conselho decidiu normatizar a prática da telenfermagem no Brasil, por meio de uma nova Resolução COFEN nº 696/2022, estabelecendo regras para a atuação em saúde digital no âmbito do Sistema Único de Saúde (SUS), bem como na saúde suplementar e privada^7^. 

A regulamentação da telenfermagem é considerada um marco histórico para a profissão, tendo em vista que contribui com a consolidação dessa prática assistencial fora do cenário de emergência pública e assegura sua aplicabilidade de modo ético e legal^8^. Dessa forma, a Resolução garante ao enfermeiro a realização da teleconsulta de forma segura e possibilita sua atuação em vários cenários.

No âmbito da Atenção Primária à Saúde (APS), a teleconsulta de enfermagem sustenta os atributos de primeiro contato, longitudinalidade, integralidade e coordenação, pois amplia o acesso dos usuários aos serviços de saúde, promovendo um atendimento viável, e que pode ser de qualidade, seguro e eficaz. Além disso, mostra-se aplicável em diversas linhas de cuidado^4^ ^),(^ ^9^. Durante a pandemia, os enfermeiros da APS mostraram competências para a realização do cuidado clínico de forma remota, sendo capazes de avaliar o estado geral de saúde, reconhecer sinais de agravamento e ofertar escuta ativa via teleatendimento, além de reconhecer e aplicar os protocolos e diretrizes clínicas^9^. 

Contudo, tais tecnologias não deveriam operar apenas como um serviço adicional da rede de atenção ou somente em períodos pandêmicos. Os benefícios das ferramentas de telessaúde para o sistema apontam para a importância de que estas sejam adotadas de forma mais abrangente no SUS e, em especial, de forma integrada à APS^10^. Diante disso, é necessário identificar como tem se dado a realização da teleconsulta de enfermagem na APS, a fim de identificar as principais habilidades para a sua realização, bem como as vantagens e limitações dessa modalidade de cuidado, visando facilitar a sua efetiva implementação. 

Frente ao exposto, o presente estudo tem como objetivo mapear as habilidades dos enfermeiros para a teleconsulta de enfermagem na Atenção Primária à Saúde. Espera-se que este estudo forneça subsídios para ampliar o conhecimento e a compreensão sobre a teleconsulta de enfermagem.

## Método

### Delineamento do estudo

Trata-se de uma revisão de escopo guiada pelas recomendações do *Joanna Briggs Institute Reviewer’s Manual* (JBI)^11^. O protocolo de pesquisa foi registrado no *Open Science Framework* (OSF) (https://osf.io/tpvbg/) e a extensão *Systematic Reviews and Meta-Analyses for Scoping Reviews* (PRISMA-ScR) foi utilizada para reportar os resultados da análise de escopo^12^.

Para a realização da revisão foram consideradas as seguintes etapas: Fase 1 - Critérios de elegibilidade; Fase 2 - Fontes de informação e busca na literatura; Fase 3 - Seleção de fontes de evidência; Fase 4 - Extração de dados; e Fase 5: Análise e apresentação dos dados.

#### Fase 1 - Critérios de elegibilidade

Os critérios de elegibilidade foram baseados principalmente nos componentes da estratégia PCC, que serviram de base para a construção da questão norteadora, a saber: População (P): refere-se aos enfermeiros; Conceito (C): teleconsulta de enfermagem; Contexto (C): Atenção Primária à Saúde. Assim, definiu-se a seguinte questão: Quais são as habilidades dos enfermeiros para a teleconsulta de enfermagem na Atenção Primária à Saúde? 

Foram considerados como critérios de inclusão: estudos publicados na íntegra, disponíveis eletronicamente, sem restrição de tempo e de idioma, cuja pesquisa apresente as habilidades dos enfermeiros para a teleconsulta de enfermagem na Atenção Primária à Saúde. Excluíram-se editoriais, cartas ao editor, vídeos, *sites*, notícias, *preprints* e resumos.

#### Fase 2 - Fontes de informação e busca na literatura

Para traçar a estratégia de busca foram utilizadas as seguintes etapas: extração, conversão, combinação, construção e uso (Figura 1)^13^. As palavras foram escolhidas a partir dos termos presentes na estratégia PCC, por meio de busca nos tesauros da área da saúde como *Medical Subject Headings* (MeSH) e Descritores em Ciências da Saúde (DeCS) e *Embase Subject Headings* (Emtree). 

**Figura 1:** Estratégia de busca. Fortaleza, CE, Brasil, 2023

Objetivo/Problema	Quais são as habilidades dos enfermeiros para a teleconsulta de enfermagem na Atenção Primária à Saúde?		
	P*	C^†^	C^‡^
**Extração**	Enfermeiro	Teleconsulta de enfermagem	Atenção Primária à Saúde
**Conversão**	*Nursing*	*Remote consultation*	*Primary Health Care*
**Combinação**	*Nursing; nurses; nurse; nursing care; Nursing, Primary; Primary Nursing Care; Care, Primary Nursing; Nursing Care, Primary*	*Remote consultation; Consultation, Remote; Teleconsultation; Teleconsultations; Asynchronous teleconsultation*	*Primary Health Care; Care Primary Health; Health Care Primary; Primary Healthcare; Primary Care;*
**Construção**	(“Nursing” OR “nurses” AND “nurse” OR “nursing care” OR “Nursing, Primary” OR “Primary Nursing Care” OR “Care, Primary Nursing” OR “Nursing Care, Primary”)	(“Remote consultation” OR “Consultation, Remote” OR “Teleconsultation” OR “Teleconsultations” OR “Asynchronous teleconsultation”)	(“Primary Health Care” OR “Care Primary Health” OR “Health Care Primary” OR “Primary Healthcare” OR “Primary Care”)
**Uso**	(“Nursing” OR “nurses” AND “nurse” OR “nursing care” OR “Nursing, Primary” OR “Primary Nursing Care” OR “Care, Primary Nursing” OR “Nursing Care, Primary”) AND (“Remote consultation” OR “Consultation, Remote” OR “Teleconsultation” OR “Teleconsultations” OR “Asynchronous teleconsultation”) AND (“Primary Health Care” OR “Care Primary Health” OR “Health Care Primary” OR “Primary Healthcare” OR “Primary Care”)		

*P = População; ^†^C = Conceito; ^‡^C = Contexto

Para tornar a busca o mais sensível possível, foram utilizados os descritores primários “*Remote consultation*”, “*Nursing Care*” e “*Primary Health Care*”, nos idiomas português e inglês. Para ampliar a busca e fazer um melhor direcionamento dos achados, foram utilizados termos sinônimos e os operadores booleanos OR e AND, adaptando os termos de acordo com a especificidade de cada base.

A estratégia de pesquisa e todo o processo de elaboração do presente trabalho adotou a metodologia de revisão sistemática de escopo proposta pelo Instituto Joanna Briggs^11^. Dessa forma, uma estratégia de pesquisa de três passos foi utilizada: I) Pesquisa inicial limitada nos bancos de dados MEDLINE/PubMed, seguida de uma análise das palavras nos títulos, resumos e termos de indexação usados para descrever o artigo; II) segunda pesquisa usando todas as palavras-chave e termos de indexação identificados nas bases de dados incluídas; e III). As referências de todos os artigos e relatórios encontrados na pesquisa foram analisadas para identificar estudos adicionais.

As bases de dados utilizadas foram: *Medical Literature Analysis and Retrieval System Online* (MEDLINE), *Cochrane Library*, *Web of Science*, Literatura Latino-Americana e do Caribe em Ciências da Saúde (LILACS), *Excerpta Medica Database* (EMBASE), *SciVerse Scopus* (SCOPUS) e *Cumulative Index to Nursing and Allied Health Literature* (CINAHL). Além disso, incluiu-se literatura cinzenta. 

As evidências da *gray literature* foram investigadas no Catálogo de Teses e Dissertações da Coordenação de Aperfeiçoamento de Pessoal de Nível Superior (CAPES), *Google* Acadêmico, pesquisas em *sites* de órgãos oficiais, manuais de instituições internacionais e nacionais, diretrizes e livros. 

A busca e a seleção dos estudos ocorreram no período de fevereiro a abril de 2023.

#### Fase 3 - Seleção de fontes de evidência 

Após a busca nas fontes de informação, os estudos foram exportados para o aplicativo *Rayyan* (*Rayyan Systems Inc, Cambridge, MA, USA*) para remoção de documentos duplicados e seleção dos estudos. Na seleção dos estudos, dois avaliadores independentes analisaram os títulos e os resumos, seguindo critérios estabelecidos para inclusão. Os textos completos dos estudos potencialmente relevantes passaram por uma análise minuciosa pelos avaliadores, mantendo os mesmos critérios de inclusão. Em casos de dúvidas ou discordâncias, um terceiro revisor especialista na área do objeto de estudo emitia seu parecer.

#### Fase 4 - Extração de dados

Para a extração dos dados, utilizou-se um instrumento padronizado elaborado previamente pelos autores, contendo informações sobre a caracterização dos estudos (título do artigo; autor(es); ano de publicação; origem/país de realização do estudo; idioma; base de dados; periódico; referência; objetivo ou questão de investigação; metodologia/métodos/tipo de estudo; população e amostra; principais resultados). 

#### Fase 5 - Análise e apresentação dos dados 

Para a compilação e apresentação dos resultados foi elaborado um quadro com as principais características dos estudos, bem como uma análise temática qualitativa foi realizada para fornecer uma visão geral da literatura sobre as habilidades dos enfermeiros para teleconsulta de enfermagem na APS. 

### Aspectos éticos

Por se tratar de uma pesquisa com dados secundários, de domínio público e disponíveis na literatura, não houve a necessidade de apreciação ética. Contudo, cabe destacar que foram respeitados os direitos autorais com correta citação e referenciamento dos estudos.

## Resultados

Primeiramente identificou-se 2068 publicações. Após a aplicação dos critérios de inclusão e leitura dos títulos e resumos, foram selecionados 1912 estudos potencialmente elegíveis, e posteriormente 1028 para serem analisados na íntegra. Desses, 23 constituíram a amostra do estudo. Para a descrição das buscas e a seleção dos estudos, utilizou-se o *Preferred Reporting Items for Systematic Review and Meta-Analyses* (PRISMA) (Figura 2). 

**Figura 2:** Fluxograma da busca nas bases de dados. Fortaleza, CE, Brasil, 2023

Os estudos identificados pertenciam em sua maioria a países do continente europeu (n=13), advindos do Reino Unido (n=5), Espanha (n=3), Suécia (n=2), Holanda (n=2) e Escócia (n=1), e de países da América (n=8), especificamente do Brasil (n=5), Canadá (n=2) e Estados Unidos (n=1). Além disso, identificou-se produções do continente asiático (n=2), especificamente de Singapura (n=1) e da Índia (n=1). 

Quanto ao delineamento dos estudos, a maior parte possuía abordagens qualitativas dos tipos: pesquisas descritivas (n= 9); relato de experiência (n=1); estudo de caso (n=1); e estudo teórico-reflexivo (n= 1). O restante das produções era de abordagem quantitativa e consistia em: ensaio clínico randomizado (n=2); coorte (n=3); e transversal (n=2). Além disso, foram identificadas três diretrizes. Na amostra final não foram incluídas dissertações e teses, pois não respondiam à questão de pesquisa.

No que concerne ao período de publicação, trata-se de artigos recentes. Houve predominância de estudos publicados nos últimos três anos (2020-2022), que totalizaram 17 produções. No período de 2012 a 2016, identificou-se quatro estudos. Já os outros dois artigos datavam de 2007 e 2009. Tais resultados reforçam o fato de que se trata de uma temática de interesse em ascensão nos últimos anos. A síntese dos artigos identificados encontra-se descrita na Figura 3.

**Figura 3:** Caracterização dos artigos identificados na revisão. Fortaleza, CE, Brasil, 2023

Número	Título	Tipo de estudo	Ano	País	Amostra
A1^14^	*Nurse practitioner-based diabetes care management: Impact of telehealth or telephone intervention on glycemic control.*	Estudo de coorte retrospectivo	2007	Estados Unidos	259
A2^15^	*Telephone consulting in primary care: a triangulated qualitative study of patients and providers.*	Estudo descritivo com abordagem qualitativa	2009	Escócia	91
A3^16^	*Nurse telephone triage in Dutch out-of-hours primary care: the relation between history taking and urgency estimation.*	Estudo de coorte	2012	Holanda	304
A4^17^	*Question design in nurse-led and GP-led telephone triage for same-day appointment requests: a comparative investigation.*	Estudo randomizado controlado	2014	Reino Unido	51
A5^18^	*Competencies required for nursing telehealth activities: A Delphi-study.*	Método Delphi	2016	Holanda	51
A6^19^	*Physical Examinations via Video for Patients With Heart Failure: Qualitative Study Using Conversation Analysis.*	Estudo descritivo com abordagem qualitativa	2020	Reino Unido	07
A7^20^	*Registered nurses´ views on telephone nursing for patients with respiratory tract infections in primary healthcare - a qualitative interview study.*	Estudo descritivo com abordagem qualitativa	2020	Suécia	12
A8^21^	*Consensus on Criteria for Good Practices in Video Consultation: A Delphi Study.*	Estudo transversal	2020	Espanha	16
A9^22^	Remote Consultations Guidance Under COVID-19* *Restrictions.*	Diretrizes	2020	Reino Unido	-
A10^23^	*Telenursing Practice Guidelines.*	Diretrizes	2020	Índia	-
A11^24^	Guia de orientação para teleconsulta de enfermagem.	Diretrizes	2020	Brasil	-
A12^25^	Implementation of remote consulting in UK primary care following the COVID-19* pandemic: a mixed-methods longitudinal study.	Estudo de coorte	2021	Reino Unido	41
A13^26^	*Video-consultation in primary health care: an implementation experience.*	Estudo descritivo com abordagem quantitativa	2021	Espanha	76
A14^27^	Implementation of COVID-19* *telemonitoring: repercussions in Nursing academic training.*	Estudo descritivo com abordagem qualitativa	2021	Brasil	17
A15^28^	Telehealth in Primary Healthcare: A Portrait of its Rapid Implementation during the COVID-19* *Pandemic.*	Estudo transversal	2021	Canadá	603
A16^29^	*Nurse-led telephone intervention for lifestyle changes on glycaemic control in people with prediabetes: Study protocol for a randomized controlled trial.*	Estudo randomizado controlado	2021	Espanha	428
A17^30^	Telemonitoring programme on COVID-19* *for a low-income community in Brazil: case study.*	Estudo descritivo com abordagem qualitativa	2021	Brasil	1076
A18^31^	Remote Consulting in Primary Health Care in Low- and Middle-Income Countries: Feasibility Study of an Online Training Program to Support Care Delivery During the COVID-19* *Pandemic.*	Estudo descritivo com abordagem qualitativa	2022	Reino Unido	75
A19^32^	*Right siting of complex acute wound management---preliminary study of teleconsultation wound services between acute and primary care in Singapore.*	Estudo descritivo com abordagem quantitativa	2022	Singapura	18
A20^33^	*Electronic Consultation by Advanced Practice Nurses to Improve Access to Specialist Care for Older Adults.*	Estudo de caso múltiplo	2022	Canadá	06
A21^34^	Relato de experiência das contribuições da telessaúde em comunidades ribeirinhas do Amazonas na pandemia.	Relato de experiência	2022	Brasil	-
A22^35^	*Saving lives by asking questions: nurses’ experiences of suicide risk assessment in telephone counselling in primary health care.*	Estudo descritivo com abordagem qualitativa	2022	Suécia	15
A23^36^	Teleconsultation as an advanced practice nursing during the COVID-19* *pandemic based on Roy and Chick-Meleis.*	Estudo teórico-reflexivo	2022	Brasil	-

*COVID-19 = *Coronavirus disease 2019*

A Figura 4 apresenta os objetivos dos estudos e os principais resultados referentes à caracterização da teleconsulta de enfermagem na Atenção Primária à Saúde.

**Figura 4:** Síntese descritiva dos estudos incluídos na revisão de escopo. Fortaleza, CE, Brasil, 2023

Número	Objetivo	Principais resultados/Conclusão
A1^14^	Comparar o impacto de programas de gerenciamento de cuidados de diabetes *mellitus* liderados por enfermeiras usando telessaúde ou intervenção por telefone.	As teleconsultas foram utilizadas para iniciar e ajustar medicamentos, solicitar exames laboratoriais, revisar e discutir resultados laboratoriais e incentivar mudanças no estilo de vida. A teleconsulta pode proporcionar melhor adesão aos planos de tratamento.
A2^15^	Compreender as perspectivas do paciente e da equipe de saúde sobre como a consulta por telefone difere da consulta presencial em termos de conteúdo, qualidade e segurança.	Embora a consulta por telefone geralmente forneça melhor acesso, a qualidade da consulta pode ser potencialmente comprometida, principalmente devido à falta de exames formais e informais. A consulta por telefone foi considerada mais adequada para acompanhamento de doenças crônicas.
A3^16^	Examinar a relação entre a abrangência da anamnese e a adequação da estimativa de urgência.	Dois tipos de perguntas podem ser distinguidos: questões discriminativas e gerais. O reconhecimento de padrões é mais importante para a identificação de problemas de saúde urgentes pelos enfermeiros do que fazer todas as perguntas cruciais durante a coleta da história.
A4^17^	Comparar a comunicação de médicos e enfermeiros com pacientes em consultas de triagem telefônica na atenção primária.	Apesar dos enfermeiros fazerem três vezes mais perguntas do que os médicos, a semelhança na duração das chamadas de triagem é explicada pelo conteúdo e a forma das perguntas usadas. Deve-se evitar perguntas projetadas para sim ou não, para que não pareça uma lista de verificação e não impossibilite a interação entre paciente e enfermeiro.
A5^18^	Identificar quais competências são necessárias para o fornecimento de telessaúde.	Habilidades de comunicação, habilidades de *coaching*, capacidade de combinar experiência clínica com telessaúde, conhecimento clínico, consciência ética e atitude de apoio foram vistas como as competências mais importantes para enfermeiros que prestam telessaúde.
A6^19^	Explorar as oportunidades e desafios do exame físico remoto de pacientes com insuficiência cardíaca usando tecnologia de comunicação mediada por vídeo.	O exame físico remoto em pacientes com insuficiência cardíaca é restrito a avaliar algumas condições, como retenção de líquidos, pressão arterial com frequência e ritmo de pulso, e saturação de oxigênio. Desse modo, os exames de vídeo são possíveis no contexto dos serviços de insuficiência cardíaca.
A7^20^	Descrever a visão de enfermeiras registradas sobre o trabalho de enfermagem por telefone com pessoas que ligam para centros de saúde primários em relação a infecções do trato respiratório.	Durante a teleconsulta, os enfermeiros devem estabelecer boa comunicação com o paciente; diferenciar problemas inofensivos de graves; lidar com as expectativas do usuário; e utilizar ferramentas de trabalho.
A8^21^	Elaborar critérios consensuais para a gestão da videoconsulta que contribuam para cuidados de saúde eficazes e de qualidade prestados pelos profissionais de saúde.	Durante a teleconsulta, devem ser levados em consideração elementos básicos, como os recursos técnicos, a relação estabelecida entre profissionais de saúde e pacientes, e o ambiente durante sua interação.
A9^22^	Apoiar a equipe de enfermagem a ver e/ou tratar pacientes por telefone ou vídeo ou por meio de outros processos de consulta remota.	A decisão de oferecer uma consulta remota em vez de uma consulta presencial deve ser baseada no processo de triagem inicial. É preciso avaliar a gravidade dos sintomas físicos e psicológicos.
A10^23^	Fornecer orientações gerais para enfermeiras registradas sobre o uso da tecnologia de telessaúde.	O uso de câmeras permite uma experiência de teleconsulta simples e barata. A comunicação durante a teleconsulta deve ser efetiva. É indispensável o registro da teleconsulta, descrevendo as etapas do processo de enfermagem.
A11^24^	Apoiar os profissionais enfermeiros na prática clínica da Teleconsulta em Enfermagem, considerando a Resolução do COFEN* número 0634/2020.	O Termo de Consentimento tem por objetivo resguardar enfermeiro e paciente, por isso sempre deve ser solicitado. É necessário dispor de sala privada e iluminada para o profissional; aparelho celular ou computador com câmera; e conexão com internet.
A12^25^	Investigar a implementação da consulta remota e explorar o impacto nos primeiros meses da pandemia de COVID-19^†^.	As enfermeiras descobriram que a consulta por telefone funcionou bem para revisões de condições crônicas, priorizando pacientes mal controlados e atendendo os pacientes presencialmente apenas para aspectos físicos.
A13^26^	Relatar a implementação da videoconsulta na APS^‡^.	Para realizar videoconsulta é necessário contar com tecnologia específica: programa para gerar reuniões virtuais, computador, *tablet* e/ou celular com câmera e microfone integrados.
A14^27^	Relatar as repercussões da implementação do monitoramento dos casos suspeitos e confirmados de COVID-19^†^ na formação acadêmica em enfermagem.	Os enfermeiros precisam ser sensíveis ao que é dito nas entrelinhas, pois a avaliação é a base para saber se o paciente precisa de cuidado direto ou é encaminhado mais adiante no sistema assistencial. É necessário realizar uma boa avaliação do estado de saúde do usuário, por meio da coleta de dados e da observação.
A15^28^	Documentar a adoção da telessaúde por vários tipos de provedores de cuidados primários de saúde durante a pandemia de COVID-19^†^ e identificar as vantagens e desvantagens da telessaúde.	Muitos enfermeiros da APS^‡^ estavam preocupados com falta de contato visual na consulta por telefone. Como alternativa para suprir o contato visual, sugeriu-se o envio de fotos. O exame físico continua a ser um elemento essencial para avaliar problemas mais complexos.
A16^29^	Avaliar a eficácia de uma intervenção telefônica liderada por enfermeiras para mudança do estilo de vida.	As evidências apontam que a teleconsulta de enfermagem na APS^‡^ tem sido satisfatória, principalmente para a avaliação de pacientes com doenças crônicas não transmissíveis, devendo ser priorizados pacientes com maior dificuldade de controle da doença.
A17^30^	Relatar o processo de criação e implementação de um programa de acompanhamento de pacientes com diagnóstico confirmado ou suspeita de COVID-19^†^ por telemonitoramento.	O programa de teleconsulta foi implantado por meio de três fases: planejamento, implementação e monitoramento. Identificou-se que o profissional precisa dispor de habilidade clínica para reconhecer sinais e sintomas de gravidade.
A18^31^	Determinar se o treinamento “consulta remota em cuidados primários de saúde” é aceitável e viável para os profissionais de saúde na zona rural da Tanzânia para apoiar a prestação de cuidados de saúde durante a pandemia.	Os profissionais expressaram que aprenderam habilidades necessárias para consultar remotamente dentro do sistema de saúde. E houve maior compreensão de tópicos, como ética em consultoria remota.
A19^32^	Fornecer evidências preliminares sobre a viabilidade do uso inaugural de teleconsulta entre hospitais e serviços de cuidados primários para tratamento de feridas agudas em Singapura.	O manejo de feridas agudas complexas é desafiador, demorado e caro. O serviço de teleconsulta de feridas é viável e aceitável, e mostrou-se eficaz para a cicatrização de feridas, além de ser potencialmente econômico.
A20^33^	Explorar como o uso do serviço *eConsult* pelos enfermeiros pode melhorar o acesso a cuidados especializados para adultos mais velhos em uma variedade de configurações.	No contexto de uma população envelhecida, o *eConsult* pode capacitar o enfermeiro para servir como um recurso humano de saúde eficaz que pode abordar os problemas de acesso únicos enfrentados por idosos em cuidados primários.
A21^34^	Relatar a criação e implementação das atividades de telessaúde desenvolvidas pelo Programa Saúde na Floresta em comunidades de áreas de conservação, no estado do Amazonas, durante a pandemia de COVID-19^†^.	Para a enfermagem, a especialidade mais solicitada foi na área de obstetrícia, seguida pela pediatria. Tais ações diminuíram a circulação dessas populações nos grandes centros urbanos, o que promoveu imunidade extra devido ao isolamento social facilitado pela utilização da telessaúde.
A22^35^	Explorar as experiências dos enfermeiros na avaliação do risco de suicídio no aconselhamento por telefone na atenção primária à saúde.	As teleconsultas não permitem exames físicos, aumentando o risco de erros, portanto, o enfermeiro deve utilizar a experiência, o conhecimento e o pensamento crítico para identificar sinais de alerta.
A23^36^	Realizar uma análise reflexiva baseada nas contribuições teóricas dos modelos de Adaptação, de Roy, e de Transição, de Chick-Meleis, e também sobre a contribuição da teleconsulta como PAE^§^ no atendimento a pacientes idosos e com doenças crônicas.	A teleconsulta é uma prática avançada de enfermagem que requer do enfermeiro o desenvolvimento pleno de seu raciocínio clínico para a implementação de um processo de enfermagem bem delineado, o qual deverá estar ancorado em um arcabouço teórico consistente.

*COFEN = Conselho Federal de Enfermagem; ^†^COVID-19 = *Coronavirus disease 2019*; ^‡^APS = Atenção Primária à Saúde; ^§^PAE = Prática Avançada de Enfermagem

Diante dos resultados, foi possível identificar diferentes tipos de habilidades necessárias para a teleconsulta de enfermagem, bem como evidências sobre as potencialidades e as barreiras da teleconsulta na APS e aspectos operacionais do processo de enfermagem na teleconsulta. Do ponto de vista qualitativo, para melhor compreensão e organização dos resultados, estes foram subdivididos em três categorias temáticas: Habilidades para teleconsulta de enfermagem na APS, Potencialidades e barreiras da teleconsulta de enfermagem na APS e Processo de enfermagem na teleconsulta. A Figura 5 apresenta as categorias temáticas. 

**Figura 5:** Categorias temáticas da revisão de escopo. Fortaleza, CE, Brasil, 2023

Categorias temáticas
Habilidades para teleconsulta de enfermagem na APS
Habilidades de comunicação^14^ ^),(^ ^16^ ^)-(^ ^25^ ^),(^ ^27^ ^)-(^ ^30^ ^),(^ ^35^, clínica^15^ ^)-(^ ^25^ ^),(^ ^27^ ^)-(^ ^28^ ^),(^ ^30^ ^),(^ ^32^ ^),(^ ^36^, tecnológica^15^ ^),(^ ^19^ ^),(^ ^21^ ^)-(^ ^24^ ^),(^ ^26^ ^)-(^ ^28^ ^),(^ ^31^ ^),(^ ^34^ ^),(^ ^36^, ética^15^ ^),(^ ^18^ ^),(^ ^21^ ^)-(^ ^24^ ^),(^ ^27^ ^)-(^ ^28^ ^),(^ ^31^ ^),(^ ^35^ ^)^ e aquelas relacionadas à infraestrutura^18^ ^)-(^ ^19^ ^),(^ ^21^ ^)-(^ ^26^ ^),(^ ^31^ ^),(^ ^33^.
**Potencialidades e barreiras da teleconsulta de enfermagem na APS**
Potencialidades - Facilidade de acesso^14^ ^),(^ ^16^ ^)-(^ ^25^ ^),(^ ^27^ ^)-(^ ^36^, acompanhamento de pacientes com COVID-19^22^ ^)-(^ ^25^ ^),(^ ^27^ ^)-(^ ^28^ ^),(^ ^30^, diabetes *mellitus* ^14^ ^),(^ ^28^ ^)-(^ ^29^, hipertensão arterial^28^, infecção do trato respiratório^20^, infecções sexualmente transmissíveis^31^, insuficiência cardíaca^19^, avaliação de feridas^25^ ^),(^ ^32^, saúde mental^35^, saúde da pessoa idosa^33^, saúde da criança^34^.
Barreiras - Falta de acesso à TIC* e/ou à internet^15^ ^),(^ ^21^ ^)-(^ ^24^ ^),(^ ^26^ ^),(^ ^33^ ^),(^ ^36^, gravidade do quadro clínico^15^ ^)-(^ ^17^ ^),(^ ^20^ ^)-(^ ^24^ ^),(^ ^27^ ^)-(^ ^28^ ^),(^ ^30^ ^),(^ ^33^ ^),(^ ^35^, não adesão do paciente^22^ ^)-(^ ^24^ ^),(^ ^33^ ^),(^ ^36^.
**Processo de enfermagem na teleconsulta**
Avaliação^16^ ^)-(^ ^17^ ^),(^ ^20^ ^)-(^ ^25^ ^),(^ ^35^, exame físico^14^ ^),(^ ^19^ ^),(^ ^24^ ^)-(^ ^25^, diagnósticos^19^ ^)-(^ ^20^ ^),(^ ^24^ ^),(^ ^36^, planejamento^24^, prescrição^23^ ^)-(^ ^25^ ^),(^ ^28^ ^),(^ ^31^ ^),(^ ^36^, evolução^22^ ^)-(^ ^24^ ^),(^ ^27^ ^),(^ ^31^ ^),(^ ^36^.

*TIC = Tecnologias da Informação e Comunicação

## Discussão

Os achados são apresentados e discutidos em três categorias temáticas: Habilidades para teleconsulta de enfermagem na APS, Potencialidades e barreiras da teleconsulta de enfermagem na APS e Processo de enfermagem na teleconsulta.

### Habilidades para teleconsulta de enfermagem na APS

Para se garantir a qualidade dos cuidados de saúde através da teleconsulta de enfermagem, os estudos apontam a necessidade de um conjunto de habilidades, dentre elas destacam-se: habilidades de comunicação, clínica, tecnológica, ética e aquelas relacionadas à infraestrutura do ambiente da teleconsulta^18^ ^),(^ ^21^ ^)-(^ ^24^ ^),(^ ^36^. 

A comunicação foi evidenciada como a principal habilidade para a realização da teleconsulta. A qualidade da interação entre profissional e paciente durante a teleconsulta é fundamental para aspectos de segurança, eficácia, experiência do paciente e, potencialmente, para resultados de saúde^17^ ^),(^ ^20^ ^),(^ ^27^. As evidências apontam que a comunicação precisa ser suficiente para identificar os sintomas do paciente e avaliar suas necessidades de saúde, sendo esta uma tarefa ainda mais difícil quando se trata de uma chamada por áudio, pois a falta de informações visuais e a necessidade de confiar apenas nas informações verbais aumentam a preocupação dos enfermeiros^20^ ^),(^ ^27^ ^)-(^ ^28^ ^),(^ ^35^. 

Outra habilidade apontada como necessária é a clínica, pois a teleconsulta requer do enfermeiro raciocínio clínico para a implementação de um processo de enfermagem bem delineado, de forma remota^20^ ^),(^ ^27^ ^),(^ ^36^. A habilidade clínica refere-se à capacidade de interpretar as respostas humanas de modo preciso para selecionar as intervenções apropriadas e avaliar o resultado alcançado, e envolve o conhecimento clínico e experiências prévias do profissional^18^ ^),(^ ^21^. 

Destaca-se a importância da habilidade clínica para o processo de interpretação e agrupamento dos dados coletados, que culmina com a tomada de decisão sobre os diagnósticos de enfermagem e que constituem a base para a seleção das ações ou intervenções com as quais se objetiva alcançar os resultados esperados^36^.

É necessário que o enfermeiro tenha habilidade tecnológica para a realização da teleconsulta, devendo conhecer noções básicas de informática, saber manusear dispositivos tecnológicos (celular, *notebook*, *tablet*), ser capaz de verificar a funcionalidade dos equipamentos de comunicação e de treinar pacientes para o uso de Tecnologias da Informação e Comunicação (TIC)^21^ ^),(^ ^28^. 

Ter habilidades e atitudes eticamente corretas como honestidade, confidencialidade, e integridade pessoal e profissional são indispensáveis durante a teleconsulta de enfermagem. O enfermeiro deve assegurar a privacidade do paciente em quaisquer circunstâncias, e garantir a confidencialidade das informações obtidas por meio da relação terapêutica^18^ ^),(^ ^26^ ^),(^ ^31^.

O enfermeiro deve garantir a privacidade e a proteção das informações, sendo o seu desrespeito uma violação da dignidade dos pacientes^18^ ^),(^ ^26^ ^),(^ ^31^. A teleconsulta implica em riscos maiores de violação de dados do que o atendimento presencial, nesse sentido, é indispensável atender às normativas vigentes, com destaque para a Lei Geral de Proteção de Dados (LGPD), nº 13.709/2018, que está em vigor desde 2020 no Brasil e vem forçando mudanças nos processos e tecnologias de cuidado^37^. 

A preparação do ambiente onde será realizada a teleconsulta de enfermagem é essencial para garantir a qualidade da consulta, devendo o enfermeiro possuir tal habilidade. Se preparado adequadamente, é possível reduzir a distância social e estabelecer maior vínculo com o paciente, tendo em vista que barreiras típicas de um ambiente de saúde, como mesa e maca de exame, não existem no cenário de uma teleconsulta, aumentando a qualidade da comunicação e a confiança entre profissionais de saúde e pacientes^21^ ^)-(^ ^22^ ^),(^ ^24^.

É essencial que, antes de iniciar a teleconsulta, seja verificada a qualidade da conexão e o funcionamento correto do áudio e/ou vídeo, para que tais fatores não comprometam a realização da consulta^22^ ^),(^ ^24^ ^),(^ ^31^. O enfermeiro deve estar preparado para casos de falha na conexão de internet, devendo manter uma atitude proativa na resolução de problemas técnicos^21^ ^),(^ ^31^.

### Potencialidades e barreiras da teleconsulta de enfermagem na APS

As evidências científicas apontam o potencial da teleconsulta de enfermagem na APS, principalmente por facilitar o acesso em áreas remotas. No entanto, para que seja realizada de forma segura e eficaz, é necessário identificar quais pacientes podem ser assistidos por essa modalidade de cuidado. Os estudos abordam a teleconsulta de enfermagem em diferentes linhas de cuidado na APS, como acompanhamento de pacientes com COVID-19, diabetes *mellitus*, hipertensão arterial, infecção do trato respiratório, infecções sexualmente transmissíveis, insuficiência cardíaca, avaliação de feridas, saúde mental, saúde da pessoa idosa, e saúde da criança^14^ ^)-(^ ^15^ ^),(^ ^19^ ^)-(^ ^20^ ^),(^ ^22^ ^)-(^ ^25^ ^),(^ ^27^ ^)-(^ ^30^ ^),(^ ^32^ ^)-(^ ^36^. 

Estudo realizado nos Estados Unidos evidenciou que a teleconsulta de enfermagem, ao ser utilizada para fornecer gerenciamento individualizado de cuidados com o diabetes, tem efeitos semelhantes no controle glicêmico quando comparada à consulta presencial^14^. Outro estudo realizado no Reino Unido apontou que a teleconsulta de enfermagem por telefone funcionou bem para revisões de condições crônicas, sendo necessário o atendimento presencial apenas para avaliação de aspectos físicos^25^.

Face às potencialidades e disseminação da teleconsulta na APS, observa-se sua aplicabilidade em pacientes com queixas e demandas diversas. Apesar das diferentes abordagens, identificou-se em comum entre os estudos que a teleconsulta de enfermagem na APS tem sido satisfatória, proporcionando melhor condição clínica ao paciente. 

Fatores como falta de acesso à TIC e/ou à internet, gravidade do quadro clínico, ou a não adesão do paciente à consulta remota impedem sua realização^22^ ^)-(^ ^24^ ^),(^ ^33^. A falta de acesso às TIC é prevalente entre a população, sendo esta uma das barreiras para a implementação da teleconsulta, por isso deve-se verificar previamente se o paciente dispõe de recursos tecnológicos suficientes e adequados para a realização da teleconsulta^15^ ^),(^ ^21^ ^)-(^ ^24^ ^),(^ ^26^ ^),(^ ^33^ ^),(^ ^36^. 

É vedado ao enfermeiro a realização de teleconsulta para atendimento de situações de urgência ou emergência^8^. Na ocasião de identificação de sinais de alerta, não se deve prosseguir com o atendimento, mas orientar a busca por um serviço de emergência^23^ ^)-(^ ^24^ ^),(^ ^33^. É responsabilidade do enfermeiro conhecer a Rede de Atenção à Saúde (RAS) disponível no território do paciente que está sendo atendido, para que, na ocorrência de qualquer necessidade de encaminhamento, possa orientar o serviço adequado para cada situação^8^.

Por ser uma modalidade de atendimento considerada recente, é comum o paciente optar por não aderir à teleconsulta, desse modo, o termo de consentimento do paciente é apontado como um requisito para a realização da teleconsulta^22^ ^)-(^ ^24^ ^),(^ ^36^. De acordo com a Resolução do COFEN nº 696/2022, que dispõe sobre a atuação da Enfermagem na Saúde Digital, normatizando a Telenfermagem, é imprescindível o consentimento do usuário/paciente envolvido ou do seu responsável legal, e que seja realizada por sua livre decisão, sendo passível de desistência a qualquer tempo e, consequentemente, a retirada do consentimento^6^.

### Processo de enfermagem na teleconsulta

A teleconsulta deve seguir o mesmo método de execução utilizado na consulta de Enfermagem presencial, considerando o Processo de Enfermagem, incluindo as etapas: avaliação de enfermagem, diagnóstico de enfermagem, planejamento de enfermagem, implementação e evolução de enfermagem^7^ ^),(^ ^38^.

Durante a avaliação de enfermagem, é recomendado que os questionamentos para a coleta de dados sejam feitos por meio de perguntas abertas e coletadas, preferencialmente, com o paciente, sendo solicitado o seu consentimento em caso de necessidade de incluir o familiar/cuidador. É preciso evitar perguntas projetadas para sim ou não, para que não pareça uma lista de verificação e não impossibilite a interação entre paciente e enfermeiro. Além disso, o uso de perguntas que induzam o paciente para uma resposta específica deve ser evitado, a fim de permitir melhor avaliação clínica^17^ ^),(^ ^21^ ^)-(^ ^22^ ^),(^ ^35^.

O fato de que paciente e profissional não compartilhem um ambiente físico impossibilita o exame físico direto e limita a avaliação^19^. Apesar disso, os estudos apontam que por meio da teleconsulta por vídeo é possível realizar a inspeção do paciente e avaliar alguns aspectos: identificar alterações na pele, presença de edema, aparência/aspecto geral, frequência respiratória, irritação ocular, força muscular e mobilidade, e presença de sangramento intenso^14^ ^),(^ ^19^ ^),(^ ^24^.

No que se refere ao tipo de chamada para a realização da teleconsulta de enfermagem, alguns dos estudos apontaram que a escolha deve ser feita de acordo com a condição clínica do paciente. Recomenda-se que alguns critérios sejam considerados, por exemplo, pacientes com casos de menor complexidade clínica geralmente podem ser atendidos por telefone^15^ ^),(^ ^26^. Já a chamada por vídeo é mais adequada para pacientes com maior complexidade e com presença de comorbidades, bem como para pacientes com deficiência auditiva^23^ ^),(^ ^26^.

Após a realização da avaliação de enfermagem, são necessárias a identificação de diagnósticos de enfermagem e a definição de um plano de cuidados para o paciente. Quando capacitado, o enfermeiro é capaz de realizar uma teleconsulta de enfermagem de qualidade, identificando diagnósticos acurados e problemas de enfermagem, com o uso de perguntas sabiamente intencionais, e a proposição de intervenções pertinentes para se atingir os resultados esperados^36^.

A evolução de enfermagem compreende a avaliação dos resultados alcançados de enfermagem e saúde da pessoa, família, coletividade e grupos especiais. Esta etapa permite a análise e a revisão de todo o Processo de Enfermagem^38^. Os estudos evidenciam que a evolução de enfermagem deve ser feita de forma detalhada e com clareza, descrevendo as etapas do processo de enfermagem^22^ ^)-(^ ^24^ ^),(^ ^27^ ^),(^ ^36^.

O processo de monitoramento reflexivo da teleconsulta é recomendado para analisar a condução da mesma, visando aperfeiçoar as próximas consultas, sendo importante identificar as principais falhas que podem ter comprometido a qualidade da consulta, desde a análise dos recursos tecnológicos como também aspectos relacionados à conduta do profissional diante do caso^21^.

Observou-se que poucos estudos abordam o processo de enfermagem durante a teleconsulta, e que, quando abordado, é feito de forma desassociada, ou seja, citando apenas alguma das etapas. Diante disso, é necessário que novos estudos sejam desenvolvidos com enfoque na sistematização da assistência de enfermagem utilizando as novas tecnologias. É também fundamental que os profissionais sejam treinados para aplicar a sistematização da assistência de enfermagem de forma remota.

A teleconsulta é uma Prática Avançada de Enfermagem (PAE) que demanda envolvimento suficiente do profissional enfermeiro para conhecer o paciente como indivíduo, por meio da aplicação de um instrumento desenvolvido e embasado em um modelo teórico que utilize taxonomias padronizadas e que seja adequado à condução da teleconsulta^36^.

Desse modo, para que a experiência de teleconsulta se torne, de fato, realidade na APS e no SUS, é necessário um investimento massivo, tanto na garantia de acesso à internet nas unidades de saúde, quanto na obtenção de computadores e telefones, fundamentais para o estabelecimento de uma comunicação satisfatória. 

Apesar do seu potencial de utilização, na maioria das vezes a teleconsulta é considerada uma oferta adicional, suplementar, não sendo utilizada pelas instituições de saúde para prestar assistência. Portanto, estimular a inserção do seu uso na prática clínica de forma rotineira, em especial na APS, e na formação dos futuros profissionais de saúde, é fundamental para atingir uma utilização adequada.

Esta revisão apontou evidências científicas de vários países cuja APS se organiza de formas diferentes, de acordo com aspectos econômicos, sociais, políticos e a partir da compreensão local do processo saúde-doença. No contexto europeu, o conceito de atenção primária pode ser entendido como um nível de atenção entre cuidado informal e cuidado hospitalar. Em contrapartida, em alguns países da América a APS refere-se a um conjunto específico de atividades de serviços de saúde voltados à população pobre. Nos países asiáticos, a reformulação da APS vem acontecendo de forma mais recente, visando um sistema acessível e equitativo. 

As contribuições deste estudo para a área da Enfermagem e saúde fundamentam-se na repercussão da teleconsulta de enfermagem nos últimos anos, principalmente em decorrência da pandemia. Estudos como este são incentivos à reflexão dos novos cenários de cuidado que o enfermeiro deve atuar, além de instigar a implementação da teleconsulta na APS.

Como limitação, pode-se citar a análise e a síntese dos dados realizadas apenas de forma descritiva. A combinação de dados de diferentes tipos de estudos é um processo desafiador que pode acarretar viés na elaboração dos resultados da revisão. Apesar das limitações, esta revisão possui como ponto de destaque o rigor metodológico requerido pelo JBI e o mapeamento de evidências sobre a teleconsulta de enfermagem. 

## Conclusão

A teleconsulta de enfermagem na atenção primária é uma forma emergente de prestar assistência à saúde. Quando resolvidas as dificuldades técnicas e organizacionais, pode economizar tempo e recursos para pacientes e profissionais, alcançando níveis satisfatórios de resolução de problemas, sem a necessidade de deslocamento às Unidades Básicas de Saúde. No entanto, para sua implementação é necessária a capacitação dos enfermeiros quanto às seguintes habilidades: comunicação, clínica, tecnológica, ética e aquelas relacionadas à infraestrutura do ambiente da teleconsulta. 

Identificou-se a necessidade de competências profissionais para lidar com a realização da teleconsulta de enfermagem, o que demanda um processo de educação permanente que perpasse os espaços de formação acadêmica, até a prática profissional do enfermeiro. 

# Translation: **Teleconsulta de enfermería en la atención primaria de salud: *scoping review*** *

**DOI:** 10.1590/1518-8345.7212.4328

**Authors:** Vitória Lídia Pereira Sousa, Francisco Wellington Dourado, Saiwori de Jesus Silva Bezerra dos Anjos, Andréa Carvalho Araújo Moreira

**Affiliations:** 1 Universidade Estadual do Ceará, Centro de Ciências da Saúde, Fortaleza, CE, Brasil.; 2 Universidade Estadual Vale do Acaraú, Centro de Ciências da Saúde, Sobral, CE, Brasil.

**Keywords:** Consulta Remota, Teleenfermería, Atención Primaria de Salud, Atención de Enfermería, Enfermería, Enfermería de Atención Primaria

## Abstract

**Objetivo:**

mapear las habilidades de los enfermeros para la teleconsulta de enfermería en la Atención Primaria de Salud.

**Método:**

esta es una revisión de alcance guiada por las recomendaciones del *Joanna Briggs Institute Reviewer’s Manual*, realizada en siete bases de datos y repositorios de tesis y disertaciones. La selección de los estudios fue realizada en *Rayyan* por dos revisores ciegos e independientes. El análisis de los datos se realizó de forma descriptiva.

**Resultados:**

se seleccionaron 23 estudios, que demostraron que las habilidades necesarias para la teleconsulta de enfermería en atención primaria fueron: comunicación, clínica, tecnológica y ética. La falta de infraestructura digital fue identificada como una de las principales barreras para la implementación de la teleconsulta. También se identificaron la falta de acceso a las tecnologías de la información y las comunicaciones y/o a internet, la gravedad del cuadro clínico y la no adherencia a la consulta remota por parte del paciente.

**Conclusión:**

la teleconsulta de enfermería en atención primaria es una forma emergente de brindar atención de salud. Sin embargo, para su implementación es necesario capacitar a los enfermeros en las siguientes habilidades: comunicación, clínica, tecnológica, ética y aquellas relacionadas con la infraestructura del ambiente de la teleconsulta.

## Destacados

**(1)** La teleconsulta de enfermería es una forma emergente de brindar atención de salud.

**(2)** La teleconsulta ahorra tiempo y recursos, alcanzando altos niveles de resolución.

**(3)** Para su implementación es necesaria la formación de enfermeros cualificados.

**(4)** Se requieren habilidades profesionales para realizar teleconsulta.

**(5)** La teleconsulta se considera una oferta complementaria a la consulta presencial.

## Introducción

La teleconsulta se puede definir como una consulta remota que incluye interacciones entre un profesional de la salud y un paciente con el objetivo de proporcionar asesoramiento diagnóstico o terapéutico de forma electrónica^1^. 

Este tipo de atención está en expansión en muchos países, teniendo como principales factores la preocupación por la reducción de los costos con cuidados de salud y los factores epidemiológicos actuales, como el envejecimiento de la población, el aumento de las enfermedades crónicas y las enfermedades infecciosas^2^.

Parece que el uso de la teleconsulta para auxiliar en la prestación de cuidados clínicos de enfermería de forma remota ha aumentado significativamente en los últimos años, con un gran potencial de aplicación en contextos de emergencia de salud pública. El contexto de pandemia de la *coronavirus disease 2019* (COVID-19) contribuyó a un cambio en el modelo de atención tradicional, los enfermeros debieron abandonar la atención rutinaria presencial e invertir en soluciones tecnológicas para realizar el seguimiento clínico no presencial de los pacientes^3^.

El uso de telesalud en los Estados Unidos aumentó durante la pandemia a aproximadamente el 50% de las visitas a la atención de salud, y la proporción debe ser de alrededor del 20% después de la pandemia, lo que corresponde a un aumento con respecto a estimaciones anteriores del 14% entre 2014 y 2020^4^. La *American Telemedicine Association* proyecta que más de la mitad de todos los servicios de salud se brindarán virtualmente para 2030, dado que los pacientes que se han adaptado a esta modalidad de cuidado esperan seguir recibiendo atención a través de ella^5^.

Una medida importante en el escenario brasileño, en el ámbito de la teleenfermería, fue la Resolución del *Conselho Federal de Enfermagem* (COFEN), que autorizó la realización de teleconsultas de enfermería durante una pandemia provocada por el nuevo *Severe Acute Respiratory Syndrome Coronavirus* 2 (SARS-CoV-2)^6^. Posteriormente, a partir de las repercusiones del uso de las tecnologías para brindar cuidados de enfermería, el citado Consejo decidió estandarizar la práctica de la teleenfermería en Brasil, por medio de una nueva Resolución COFEN nº 696/2022, estableciendo reglas de actuación en salud digital en el ámbito del SUS, así como en salud suplementaria y privada^7^. 

La regulación de la teleenfermería es considerada un hito histórico para la profesión, ya que contribuye a la consolidación de esta práctica de cuidado fuera del escenario de emergencia pública y asegura su aplicabilidad de manera ética y legal^8^. De este modo, la Resolución garantiza que el enfermero pueda realizar teleconsultas de forma segura y le permite actuar en diversos escenarios.

En el ámbito de la Atención Primaria de Salud (APS), la teleconsulta de enfermería apoya los atributos de primer contacto, longitudinalidad, integralidad y coordinación, en la medida que amplía el acceso de los usuarios a los servicios de salud, promoviendo una atención viable, y que puede ser de calidad, segura y eficaz. Además, es aplicable en diferentes líneas de cuidado^4^ ^),(^ ^9^. Durante la pandemia, los enfermeros de la APS demostraron habilidades para brindar cuidado clínico remoto, pudiendo evaluar el estado general de salud, reconocer signos de empeoramiento y ofrecer escucha activa vía teleasistencia, además de reconocer y aplicar protocolos y guías clínicas^9^. 

Sin embargo, dichas tecnologías no deberían funcionar únicamente como un servicio adicional a la red de atención o solamente durante períodos de pandemia. Los beneficios de las herramientas de telesalud para el sistema señalan la importancia de su adopción más integral en el *Sistema Único de Saúde* (SUS) y, en particular, de forma integrada con la APS^10^. Ante esto, es necesario identificar cómo se ha realizado la teleconsulta de enfermería en la APS, con el fin de identificar las principales habilidades para realizarla, así como las ventajas y limitaciones de este tipo de cuidado, con el objetivo de facilitar su efectiva implementación. 

Teniendo en cuenta lo anterior, el presente estudio tiene como objetivo mapear las habilidades de los enfermeros para la teleconsulta de enfermería en la Atención Primaria de Salud. Se espera que este estudio proporcione subsidios para ampliar el conocimiento y la comprensión sobre la teleconsulta de enfermería. 

## Método

### Diseño del estudio

Esta es una revisión de alcance guiada por las recomendaciones del *Joanna Briggs Institute Reviewer’s Manual* (JBI)^11^. El protocolo de investigación se registró en el *Open Science Framework* (OSF) (https://osf.io/tpvbg/) y se utilizó la extensión *Systematic Reviews and Meta-Analyses for Scoping Reviews* (PRISMA-ScR) para informar los resultados del análisis de alcance^12^.

Para realizar la revisión se consideraron los siguientes pasos: Fase 1 - Criterios de elegibilidad; Fase 2 - Fuentes de información y búsqueda en la literatura; Fase 3 - Selección de fuentes de evidencia; Fase 4 - Extracción de datos; y Fase 5: Análisis y presentación de los datos.

#### Fase 1 - Criterios de elegibilidad

Los criterios de elegibilidad se basaron principalmente en los componentes de la estrategia PCC, que sirvieron de base para la construcción de la pregunta orientadora, a saber: Población (P): se refiere a los enfermeros; Concepto (C): teleconsulta de enfermería; Contexto (C): Atención Primaria de Salud. Así, se definió la siguiente pregunta: ¿Cuáles son las competencias de los enfermeros para la teleconsulta de enfermería en la Atención Primaria de Salud? 

Los criterios de inclusión fueron: estudios publicados íntegramente, disponibles electrónicamente, sin restricciones de tiempo ni de idioma, cuyas investigaciones presenten las habilidades de los enfermeros para la teleconsulta de enfermería en la Atención Primaria de Salud. Se excluyeron editoriales, cartas al editor, videos, sitios web, noticias, *preprints* y resúmenes.

#### Fase 2 - Fuentes de información y búsqueda en la literatura

Para delinear la estrategia de búsqueda se utilizaron los siguientes pasos: extracción, conversión, combinación, construcción y uso (Figura 1)^13^. Las palabras fueron elegidas con base en los términos presentes en la estrategia PCC, mediante una búsqueda en tesauros de salud como *Medical Subject Headings* (MeSH) y Descriptores en Ciencias de la Salud (DeCS) y *Embase Subject Headings* (Emtree).

**Figura 1:** Estrategia de búsqueda. Fortaleza, CE, Brasil, 2023

Objetivo/Problema	¿Cuáles son las habilidades de los enfermeros para la teleconsulta de enfermería en la Atención Primaria de Salud?		
	P*	C^†^	C^‡^
**Extracción**	Enfermero	Teleconsulta de enfermería	Atención Primaria de Salud
**Conversión**	*Nursing*	*Remote consultation*	*Primary Health Care*
**Combinación**	*Nursing; nurses; nurse; nursing care; Nursing, Primary; Primary Nursing Care; Care, Primary Nursing; Nursing Care, Primary*	*Remote consultation; Consultation, Remote; Teleconsultation; Teleconsultations; Asynchronous teleconsultation*	*Primary Health Care; Care Primary Health; Health Care Primary; Primary Healthcare; Primary Care;*
**Construcción**	(“Nursing” OR “nurses” AND “nurse” OR “nursing care” OR “Nursing, Primary” OR “Primary Nursing Care” OR “Care, Primary Nursing” OR “Nursing Care, Primary”)	(“Remote consultation” OR “Consultation, Remote” OR “Teleconsultation” OR “Teleconsultations” OR “Asynchronous teleconsultation”)	(“Primary Health Care” OR “Care Primary Health” OR “Health Care Primary” OR “Primary Healthcare” OR “Primary Care”)
**Uso**	(“Nursing” OR “nurses” AND “nurse” OR “nursing care” OR “Nursing, Primary” OR “Primary Nursing Care” OR “Care, Primary Nursing” OR “Nursing Care, Primary”) AND (“Remote consultation” OR “Consultation, Remote” OR “Teleconsultation” OR “Teleconsultations” OR “Asynchronous teleconsultation”) AND (“Primary Health Care” OR “Care Primary Health” OR “Health Care Primary” OR “Primary Healthcare” OR “Primary Care”)		

*P = Población; ^†^C = Concepto; ^‡^C = Contexto

Para hacer la búsqueda lo más sensible posible, se utilizaron los descriptores primarios “*Remote consultation*”, “*Nursing Care*” y “*Primary Health Care*”, en portugués e inglés. Para ampliar la búsqueda y orientar mejor los hallazgos se utilizaron términos sinónimos y los operadores booleanos OR y AND, adaptando los términos según la especificidad de cada base.

La estrategia de investigación y todo el proceso de elaboración de este trabajo adoptó la metodología de revisión sistemática de alcance propuesta por el *Instituto Joanna Briggs* ^11^. Por lo tanto, se utilizó una estrategia de búsqueda de tres pasos: I) Búsqueda inicial limitada en las bases de datos MEDLINE/PubMed, seguida de un análisis de las palabras en los títulos, resúmenes y términos de indexación utilizados para describir el artículo; II) Segunda búsqueda utilizando todas las palabras clave y términos de indexación identificados en las bases de datos incluidas; y III) Se analizaron las referencias de todos los artículos e informes encontrados en la búsqueda para identificar estudios adicionales.

Las bases de datos utilizadas fueron: *Medical Literature Analysis and Retrieval System Online* (MEDLINE), *Cochrane Library*, *Web of Science*, Literatura Latinoamericana y del Caribe en Ciencias de la Salud (LILACS), *Excerpta Medica Database* (EMBASE), *SciVerse Scopus* (SCOPUS) y *Cumulative Index to Nursing and Allied Health Literature* (CINAHL). Además, se incluyó literatura gris. 

La evidencia de *gray literature* fue investigada en el Catálogo de Tesis y Disertaciones de la *Coordenação de Aperfeiçoamento de Pessoal de Nível Superior* (CAPES), *Google* Académico, búsquedas en sitios web de organismos oficiales, manuales de instituciones internacionales y nacionales, directrices y libros. 

La búsqueda y la selección de los estudios se realizaron de febrero a abril de 2023.

#### Fase 3 - Selección de fuentes de evidencia 

Después de buscar fuentes de información, los estudios se exportaron a la aplicación *Rayyan* (*Rayyan Systems Inc, Cambridge, MA, USA*) para eliminar documentos duplicados y seleccionar los estudios. Al seleccionar los estudios, dos evaluadores independientes analizaron los títulos y los resúmenes, siguiendo los criterios de inclusión establecidos. Los textos completos de los estudios potencialmente relevantes fueron sometidos a un análisis exhaustivo por parte de los evaluadores, manteniendo los mismos criterios de inclusión. En casos de dudas o desacuerdos, un tercer revisor especializado en el área del objeto de estudio emitía su opinión.

#### Fase 4 - Extracción de datos

Para la extracción de los datos se utilizó un instrumento estandarizado previamente elaborado por los autores, que contiene información sobre la caracterización de los estudios (título del artículo; autor(es); año de publicación; origen/país del estudio realizado; idioma; base de datos; periódico; referencia; objetivo o pregunta de investigación; metodología/métodos/tipo de estudio; población y muestra; principales resultados). 

#### Fase 5 - Análisis y presentación de los datos 

Para recopilar y presentar los resultados, se creó una tabla con las principales características de los estudios, así como se realizó un análisis temático cualitativo para brindar una visión general de la literatura sobre las habilidades de los enfermeros para la teleconsulta de enfermería en la APS. 

### Aspectos éticos

Como se trataba de una investigación que utilizó datos secundarios, de dominio público y disponibles en la literatura, no hubo necesidad de una evaluación ética. Sin embargo, cabe destacar que se respetaron los derechos de autor con la correcta citación y referenciación de los estudios.

## Resultados

En primer lugar, se identificaron 2068 publicaciones. Tras aplicar los criterios de inclusión y leer los títulos y resúmenes, se seleccionaron 1.912 estudios potencialmente elegibles, y posteriormente 1.028 para ser analizados en su totalidad. De ellos, 23 constituyeron la muestra de estudio. Para describir las búsquedas y seleccionar los estudios se utilizó el *Preferred Reporting Items for Systematic Review and Meta-Analyses* (PRISMA) (Figura 2). 

**Figura 2:** Flujograma de la búsqueda en las bases de datos. Fortaleza, CE, Brasil, 2023

Los estudios identificados pertenecían en su mayoría a países del continente europeo (n=13), procedentes de Reino Unido (n=5), España (n=3), Suecia (n=2), Holanda (n=2) y Escocia (n=1), y de países de América (n=8), específicamente Brasil (n=5), Canadá (n=2) y Estados Unidos (n=1). Además, se identificaron producciones del continente asiático (n=2), concretamente de Singapur (n=1) y de India (n=1). 

En cuanto al diseño de los estudios, la mayoría tuvo enfoques cualitativos de los siguientes tipos: investigación descriptiva (n= 09); relato de experiencia (n=01); estudio de caso (n=01); y estudio teórico-reflexivo (n= 01). El resto de las producciones tuvieron un enfoque cuantitativo y consistieron en: ensayo clínico randomizado (n=2); cohorte (n=3); y transversal (n=2). Asimismo, se identificaron tres directrices. Las disertaciones y tesis no se incluyeron en la muestra final, ya que no respondían a la pregunta de investigación.

En cuanto al periodo de publicación, se trata de artículos recientes. Hubo predominio de estudios publicados en los últimos tres años (2020-2022), que sumaron 17 producciones. En el periodo de 2012 a 2016 se identificaron 4 estudios. Los otros dos artículos databan de 2007 y 2009. Estos resultados refuerzan el hecho de que se trata de un tema de creciente interés en los últimos años. La síntesis de los artículos identificados se describe en la Figura 3.

**Figura 3:** Caracterización de los artículos identificados en la revisión. Fortaleza, CE, Brasil, 2023

Número	Título	Tipo de estudio	Año	País	Muestra
A1^14^	*Nurse practitioner-based diabetes care management: Impact of telehealth or telephone intervention on glycemic control.*	Estudio de cohorte retrospectivo	2007	Estados Unidos	259
A2^15^	*Telephone consulting in primary care: a triangulated qualitative study of patients and providers.*	Estudio descriptivo con enfoque cualitativo	2009	Escocia	91
A3^16^	*Nurse telephone triage in Dutch out-of-hours primary care: the relation between history taking and urgency estimation.*	Estudio de cohorte	2012	Holanda	304
A4^17^	*Question design in nurse-led and GP-led telephone triage for same-day appointment requests: a comparative investigation.*	Estudio controlado randomizado	2014	Reino Unido	51
A5^18^	*Competencies required for nursing telehealth activities: A Delphi-study.*	Método Delphi	2016	Holanda	51
A6^19^	*Physical Examinations via Video for Patients With Heart Failure: Qualitative Study Using Conversation Analysis.*	Estudio descriptivo con enfoque cualitativo	2020	Reino Unido	07
A7^20^	*Registered nurses´ views on telephone nursing for patients with respiratory tract infections in primary healthcare - a qualitative interview study.*	Estudio descriptivo con enfoque cualitativo	2020	Suecia	12
A8^21^	*Consensus on Criteria for Good Practices in Video Consultation: A Delphi Study.*	Estudio transversal	2020	España	16
A9^22^	Remote Consultations Guidance Under COVID-19* *Restrictions.*	Directrices	2020	Reino Unido	-
A10^23^	*Telenursing Practice Guidelines.*	Directrices	2020	India	-
A11^24^	*Guia de orientação para teleconsulta de enfermagem*.	Directrices	2020	Brasil	-
A12^25^	Implementation of remote consulting in UK primary care following the COVID-19* *pandemic: a mixed-methods longitudinal study.*	Estudio de cohorte	2021	Reino Unido	41
A13^26^	*Video-consultation in primary health care: an implementation experience.*	Estudio descriptivo con enfoque cuantitativo	2021	España	76
A14^27^	Implementation of COVID-19* *telemonitoring: repercussions in Nursing academic training.*	Estudio descriptivo con enfoque cualitativo	2021	Brasil	17
A15^28^	Telehealth in Primary Healthcare: A Portrait of its Rapid Implementation during the COVID-19* *Pandemic.*	Estudio transversal	2021	Canadá	603
A16^29^	*Nurse-led telephone intervention for lifestyle changes on glycaemic control in people with prediabetes: Study protocol for a randomized controlled trial.*	Estudio controlado randomizado	2021	España	428
A17^30^	Telemonitoring programme on COVID-19* *for a low-income community in Brazil: case study.*	Estudio descriptivo con enfoque cualitativo	2021	Brasil	1076
A18^31^	Remote Consulting in Primary Health Care in Low- and Middle-Income Countries: Feasibility Study of an Online Training Program to Support Care Delivery During the COVID-19* *Pandemic.*	Estudio descriptivo con enfoque cualitativo	2022	Reino Unido	75
A19^32^	*Right siting of complex acute wound management---preliminary study of teleconsultation wound services between acute and primary care in Singapore.*	Estudio descriptivo con enfoque cuantitativo	2022	Singapur	18
A20^33^	*Electronic Consultation by Advanced Practice Nurses to Improve Access to Specialist Care for Older Adults.*	Estudio de caso múltiple	2022	Canadá	06
A21^34^	*Relato de experiência das contribuições da telessaúde em comunidades ribeirinhas do Amazonas na pandemia.*	Relato de experiencia	2022	Brasil	-
A22^35^	*Saving lives by asking questions: nurses’ experiences of suicide risk assessment in telephone counselling in primary health care.*	Estudio descriptivo con enfoque cualitativo	2022	Suecia	15
A23^36^	Teleconsultation as an advanced practice nursing during the COVID-19* *pandemic based on Roy and Chick-Meleis.*	Estudio teórico-reflexivo	2022	Brasil	-

*COVID-19 = *Coronavirus disease 2019*

La Figura 4 presenta los objetivos de los estudios y los principales resultados en cuanto a la caracterización de la teleconsulta de enfermería en la Atención Primaria de Salud.

**Figura 4:** Síntesis descriptiva de los estudios incluidos en la revisión de alcance. Fortaleza, CE, Brasil, 2023

Número	Objetivo	Principales resultados/Conclusión
A1^14^	Comparar el impacto de programas de gestión del cuidado de diabetes *mellitus* dirigidos por enfermeras mediante telesalud o intervención telefónica.	Se utilizaron teleconsultas para iniciar y ajustar medicamentos, solicitar pruebas de laboratorio, revisar y discutir resultados de laboratorio y fomentar cambios en el estilo de vida. La teleconsulta puede proporcionar una mejor adherencia a los planes de tratamiento.
A2^15^	Comprender las perspectivas del paciente y del equipo de salud sobre en qué se diferencia una consulta telefónica de una consulta en persona en términos de contenido, calidad y seguridad.	Aunque la consulta telefónica generalmente proporciona un mejor acceso, la calidad de la consulta puede verse potencialmente comprometida, particularmente debido a la falta de exámenes formales e informales. La consulta telefónica se consideró más apropiada para el seguimiento de enfermedades crónicas.
A3^16^	Examinar la relación entre la exhaustividad de la anamnesis y la adecuación de la estimación de urgencia.	Se pueden distinguir dos tipos de preguntas: las discriminativas y las generales. El reconocimiento de patrones es más importante para que los enfermeros identifiquen problemas de salud urgentes que formular todas las preguntas cruciales durante la anamnesis.
A4^17^	Comparar la comunicación de médicos y enfermeros con los pacientes en consultas telefónicas de triaje en atención primaria.	Aunque los enfermeros hacen tres veces más preguntas que los médicos, la similitud en la duración de las llamadas de triaje se explica por el contenido y la forma de las preguntas utilizadas. Se deben evitar las preguntas diseñadas para sí o no, para que no parezca una lista de verificación y no imposibilite la interacción entre paciente y enfermero.
A5^18^	Identificar qué habilidades se necesitan para brindar telesalud.	Las habilidades de comunicación, las habilidades de *coaching*, la capacidad de combinar experiencia clínica con telesalud, el conocimiento clínico, la conciencia ética y la actitud de apoyo se consideraron las competencias más importantes de los enfermeros que brindan telesalud.
A6^19^	Explorar las oportunidades y desafíos del examen físico remoto de pacientes con insuficiencia cardíaca utilizando tecnología de comunicación mediada por video.	El examen físico remoto en pacientes con insuficiencia cardíaca se limita a evaluar ciertas condiciones, como la retención de líquidos, la presión arterial y el ritmo del pulso, y la saturación de oxígeno. Por lo tanto, los exámenes por vídeo son posibles en el contexto de los servicios de insuficiencia cardíaca.
A7^20^	Describir las opiniones de las enfermeras registradas sobre el trabajo de enfermería telefónica con personas que llaman a los centros de atención primaria de salud con relación a infecciones del tracto respiratorio.	Durante la teleconsulta, las enfermeras deben establecer una buena comunicación con el paciente; diferenciar los problemas inofensivos de los graves; manejar las expectativas de los usuarios; y utilizar herramientas de trabajo.
A8^21^	Desarrollar criterios consensuados para la gestión de la videoconsulta que contribuyan para cuidados de salud eficaces y de calidad brindados por los profesionales de la salud.	Durante la teleconsulta se deben tener en cuenta elementos básicos, como los recursos técnicos, la relación que se establece entre los profesionales de la salud y los pacientes, y el entorno durante su interacción.
A9^22^	Apoyar al personal de enfermería para ver y/o tratar a los pacientes por teléfono o video o mediante otros procesos de consulta remota.	La decisión de ofrecer una consulta remota en lugar de una consulta en persona debe basarse en el proceso de triaje inicial. Es necesario evaluar la gravedad de los síntomas físicos y psicológicos.
A10^23^	Proporcionar orientación general a las enfermeras registradas sobre el uso de la tecnología de telesalud.	El uso de cámaras permite una experiencia de teleconsulta sencilla y económica. La comunicación durante la teleconsulta debe ser efectiva. Es fundamental registrar la teleconsulta, describiendo las etapas del proceso de enfermería.
A11^24^	Apoyar a los profesionales de enfermería en la práctica clínica de la Teleconsulta en Enfermería, considerando la Resolución COFEN* número 0634/2020.	El Formulario de Consentimiento tiene como objetivo proteger a enfermeros y pacientes, por lo que siempre debe solicitarse. Es necesario disponer de una sala privada y luminosa para el profesional; teléfono celular o computadora con cámara; y conexión a internet.
A12^25^	Investigar la implementación de la consulta remota y explorar el impacto en los primeros meses de la pandemia de COVID-19^†^.	Las enfermeras descubrieron que la consulta telefónica funcionaba bien para las revisiones de enfermedades crónicas, dando prioridad a los pacientes mal controlados y atendiendo presencialmente a los pacientes solamente para aspectos físicos.
A13^26^	Reportar la implementación de la videoconsulta en la APS^‡^.	Para realizar una videoconsulta es necesario contar con tecnología específica: un programa para generar reuniones virtuales, una computadora, *tablet* y/o celular con cámara y micrófono integrados.
A14^27^	Informar sobre las repercusiones de la implementación del seguimiento de casos sospechosos y confirmados de COVID-19^†^ en la formación académica de enfermería.	Las enfermeras deben ser sensibles a lo que se dice entre líneas, ya que la evaluación es la base para saber si el paciente necesita cuidado directo o si es remitido a otra parte del sistema de atención. Es necesario realizar una buena valoración del estado de salud del usuario, mediante la recolección de datos y la observación.
A15^28^	Documentar la adopción de la telesalud por parte de varios tipos de proveedores de cuidado primario de salud durante la pandemia de COVID-19^†^ e identificar las ventajas y desventajas de la telesalud.	Muchos enfermeros de la APS^‡^ estaban preocupados por la falta de contacto visual durante las consultas telefónicas. Como alternativa a brindar contacto visual, se sugirió enviar fotografías. El examen físico sigue siendo un elemento esencial en la evaluación de problemas más complejos.
A16^29^	Evaluar la efectividad de una intervención telefónica dirigida por enfermeras para cambio de estilo de vida.	La evidencia indica que la teleconsulta de enfermería en la APS^‡^ ha sido satisfactoria, especialmente para la evaluación de pacientes con enfermedades crónicas no transmisibles, debiendo priorizarse a los pacientes con mayor dificultad para controlar la enfermedad.
A17^30^	Informar sobre el proceso de creación e implementación de un programa de seguimiento de pacientes con diagnóstico confirmado o sospecha de COVID-19^†^ por telemonitoreo.	El programa de teleconsulta se implementó por medio de tres fases: planificación, implementación y seguimiento. Se identificó que el profesional necesita tener habilidades clínicas para reconocer signos y síntomas de gravedad.
A18^31^	Determinar si la capacitación en “consultas remotas en atención primaria de salud” es aceptable y factible para que los profesionales de la salud en las zonas rurales de Tanzania apoyen la prestación de cuidados de salud durante la pandemia.	Los profesionales expresaron que aprendieron habilidades necesarias para realizar consultas remotas dentro del sistema de salud. Y hubo una mayor comprensión de temas como la ética en la consultoría remota.
A19^32^	Proporcionar evidencia preliminar sobre la viabilidad del uso inaugural de la teleconsulta entre hospitales y servicios de atención primaria para el cuidado de heridas agudas en Singapur.	El tratamiento de heridas agudas complejas es un desafío, requiere mucho tiempo y es costoso. El servicio de teleconsulta de heridas es viable y aceptable, y ha demostrado ser eficaz para la cicatrización de heridas, además de ser potencialmente económico.
A20^33^	Explorar cómo el uso del servicio *eConsult* por parte de los enfermeros puede mejorar el acceso a cuidados especializados para adultos mayores en una variedad de entornos.	En el contexto de una población que envejece, *eConsult* puede capacitar al enfermero para que actúe como un recurso humano de salud eficaz que pueda abordar los problemas de acceso únicos que enfrentan los adultos mayores en cuidados primarios.
A21^34^	Reportar la creación e implementación de actividades de telesalud desarrolladas por el *Programa Saúde na Floresta* en comunidades de áreas de conservación, en el estado de Amazonas, durante la pandemia de COVID-19^†^.	Para enfermería, la especialidad más solicitada fue el área de obstetricia, seguida de pediatría. Tales acciones redujeron la circulación de estas poblaciones en los grandes centros urbanos, lo que promovió una inmunidad extra debido al aislamiento social facilitado por el uso de la telesalud.
A22^35^	Explorar las experiencias de los enfermeros en la evaluación del riesgo de suicidio en el asesoramiento telefónico en la atención primaria de salud.	Las teleconsultas no permiten realizar exámenes físicos, aumentando el riesgo de errores, por lo que el enfermero debe utilizar la experiencia, el conocimiento y el pensamiento crítico para identificar señales de alerta.
A23^36^	Realizar un análisis reflexivo basado en los aportes teóricos de los modelos de Adaptación, de Roy, y de Transición, de Chick-Meleis, y también en el aporte de la teleconsulta como PAE^§^ en la atención a pacientes mayores y con enfermedades crónicas.	La teleconsulta es una práctica avanzada de enfermería que requiere que el enfermero desarrolle plenamente su razonamiento clínico para implementar un proceso de enfermería bien diseñado, que debe estar anclado en un marco teórico consistente.

*COFEN = *Conselho Federal de Enfermagem*; ^†^COVID-19 = *Coronavirus disease 2019*; ^‡^APS = Atención Primaria de Salud; ^§^PAE = Práctica Avanzada de Enfermería

Teniendo en cuenta los resultados, fue posible identificar diferentes tipos de habilidades necesarias para la teleconsulta de enfermería, así como evidencia sobre las potencialidades y las barreras de la teleconsulta en la APS y aspectos operativos del proceso de enfermería en teleconsulta. Desde el punto de vista cualitativo, para una mejor comprensión y organización de los resultados, fueron subdivididos en tres categorías temáticas: Habilidades para la teleconsulta de enfermería en la APS, Potencialidades y barreras de la teleconsulta de enfermería en la APS y Proceso de enfermería en la teleconsulta. La Figura 5 presenta las categorías temáticas. 

**Figura 5:** Categorías temáticas de la revisión de alcance. Fortaleza, CE, Brasil, 2023

Categorías temáticas
Habilidades para la teleconsulta de enfermería en la APS
Habilidades de comunicación^14^ ^),(^ ^16^ ^)-(^ ^25^ ^),(^ ^27^ ^)-(^ ^30^ ^),(^ ^35^, clínica^15^ ^)-(^ ^25^ ^),(^ ^27^ ^)-(^ ^28^ ^),(^ ^30^ ^),(^ ^32^ ^),(^ ^36^, tecnológica^15^ ^),(^ ^19^ ^),(^ ^21^ ^)-(^ ^24^ ^),(^ ^26^ ^)-(^ ^28^ ^),(^ ^31^ ^),(^ ^34^ ^),(^ ^36^, ética^15^ ^),(^ ^18^ ^),(^ ^21^ ^)-(^ ^24^ ^),(^ ^27^ ^)-(^ ^28^ ^),(^ ^31^ ^),(^ ^35^ y aquellas relacionadas con la infraestructura^18^ ^)-(^ ^19^ ^),(^ ^21^ ^)-(^ ^26^ ^),(^ ^31^ ^),(^ ^33^.
**Potencialidades y barreras de la teleconsulta de enfermería en la APS**
Potencialidades - Facilidad de acceso^14^ ^),(^ ^16^ ^)-(^ ^25^ ^),(^ ^27^ ^)-(^ ^36^, seguimiento de pacientes con COVID-19^22^ ^)-(^ ^25^ ^),(^ ^27^ ^)-(^ ^28^ ^),(^ ^30^, diabetes *mellitus* ^14^ ^),(^ ^28^ ^)-(^ ^29^, hipertensión arterial^28^, infección del tracto respiratorio^20^, infecciones de transmisión sexual^31^, insuficiencia cardíaca^19^, evaluación de heridas^25^ ^,^ ^32^, salud mental^35^, salud de la persona mayor^33^, salud infantil^34^.
Barreras - Falta de acceso a las TIC* y/o a internet^15^ ^),(^ ^21^ ^)-(^ ^24^ ^),(^ ^26^ ^),(^ ^33^ ^),(^ ^36^, gravedad del cuadro clínico^15^ ^)-(^ ^17^ ^),(^ ^20^ ^)-(^ ^24^ ^),(^ ^27^ ^)-(^ ^28^ ^),(^ ^30^ ^),(^ ^33^ ^),(^ ^35^, no adherencia del paciente^22^ ^)-(^ ^24^ ^),(^ ^33^ ^),(^ ^36^.
**Proceso de enfermería en la teleconsulta**
Evaluación^16^ ^)-(^ ^17^ ^),(^ ^20^ ^)-(^ ^25^ ^),(^ ^35^, examen físico^14^ ^),(^ ^19^ ^),(^ ^24^ ^)-(^ ^25^, diagnósticos^19^ ^)-(^ ^20^ ^),(^ ^24^ ^),(^ ^36^, planificación^24^, prescripción^23^ ^)-(^ ^25^ ^),(^ ^28^ ^),(^ ^31^ ^),(^ ^36^, evolución^22^ ^)-(^ ^24^ ^),(^ ^27^ ^),(^ ^31^ ^),(^ ^36^.

*TIC = Tecnologías de la Información y las Comunicaciones

## Discusión

Los hallazgos se presentan y discuten en tres categorías temáticas: Habilidades para la teleconsulta de enfermería en la APS, Potencialidades y barreras de la teleconsulta de enfermería en la APS y Proceso de enfermería en la teleconsulta.

### Habilidades para la teleconsulta de enfermería en la APS

Para garantizar la calidad de la atención en salud a través de la teleconsulta de enfermería, los estudios señalan la necesidad de un conjunto de habilidades, entre las que se destacan: habilidades de comunicación, clínica, tecnológica, ética y aquellas relacionadas con la infraestructura del ambiente de la teleconsulta^18^ ^),(^ ^21^ ^)-(^ ^24^ ^),(^ ^36^. 

Se destacó la comunicación como la principal habilidad para realizar la teleconsulta. La calidad de la interacción entre profesional y paciente durante la teleconsulta es fundamental para aspectos de seguridad, eficacia, experiencia del paciente y, potencialmente, resultados de salud^17^ ^),(^ ^20^ ^),(^ ^27^. La evidencia sugiere que la comunicación debe ser suficiente para identificar los síntomas del paciente y evaluar sus necesidades de salud, siendo esta una tarea aún más difícil cuando se trata de una llamada de audio, ya que la falta de información visual y la necesidad de confiar únicamente en la información verbal aumentan la preocupación de los enfermeros^20^ ^),(^ ^27^ ^)-(^ ^28^ ^),(^ ^35^. 

Otra habilidad identificada como necesaria es la clínica, ya que la teleconsulta requiere que las enfermeras tengan razonamiento clínico para implementar un proceso de enfermería bien diseñado, de forma remota^20^ ^),(^ ^27^ ^),(^ ^36^. La habilidad clínica se refiere a la capacidad de interpretar las respuestas humanas con precisión para seleccionar intervenciones apropiadas y evaluar el resultado logrado, e involucra el conocimiento clínico y las experiencias previas del profesional^18^ ^),(^ ^21^. 

Se destaca la importancia de las habilidades clínicas en el proceso de interpretación y agrupación de los datos recolectados, que culmina con la toma de decisiones sobre los diagnósticos de enfermería y que constituyen la base para seleccionar las acciones o intervenciones con las cuales se plantea alcanzar los resultados esperados^36^.

Es necesario que el enfermero tenga habilidad tecnológica para realizar la teleconsulta, debiendo tener conocimientos básicos de informática, saber manejar dispositivos tecnológicos (celular, *notebook*, *tablet*), ser capaz de comprobar la funcionalidad de los equipos de comunicación y capacitar a los pacientes para utilizar las Tecnologías de la Información y las Comunicaciones (TIC)^21^ ^),(^ ^28^. 

Tener habilidades y actitudes éticamente correctas como honestidad, confidencialidad e integridad personal y profesional son fundamentales durante la teleconsulta de enfermería. El enfermero debe asegurar la privacidad del paciente en cualquier circunstancia, y garantizar la confidencialidad de la información obtenida por medio de la relación terapéutica^18^ ^),(^ ^26^ ^),(^ ^31^.

El enfermero debe garantizar la privacidad y la protección de la información, siendo la falta de respeto una violación a la dignidad de los pacientes^18^ ^),(^ ^26^ ^),(^ ^31^. La teleconsulta implica mayores riesgos de vulneración de datos que la atención presencial, en este sentido, es fundamental cumplir con la normativa vigente, con énfasis en la *Lei Geral de Proteção de Dados* (LGPD), nº 13.709/2018, vigente desde 2020 en Brasil y que viene forzando cambios en los procesos y tecnologías de cuidado^37^. 

Preparar el ambiente donde se realizará la teleconsulta de enfermería es fundamental para garantizar la calidad de la consulta, y el enfermero debe tener esta habilidad. Si se prepara adecuadamente, es posible reducir la distancia social y establecer un mayor vínculo con el paciente, considerando que las barreras propias de un ambiente de salud, como mesa y camilla de examen, no existen en el escenario de una teleconsulta, aumentando la calidad de la comunicación y la confianza entre profesionales de la salud y pacientes^21^ ^)-(^ ^22^ ^),(^ ^24^.

Es imprescindible que, antes de iniciar la teleconsulta, se compruebe la calidad de la conexión y el correcto funcionamiento del audio y/o vídeo, para que dichos factores no comprometan la consulta^22^ ^),(^ ^24^ ^),(^ ^31^. El enfermero debe estar preparado en casos de fallo de conexión a internet, y mantener una actitud proactiva a la hora de resolver problemas técnicos^21^ ^),(^ ^31^.

### Potencialidades y barreras de la teleconsulta de enfermería en la APS

La evidencia científica apunta al potencial de la teleconsulta de enfermería en la APS, principalmente porque facilita el acceso en zonas remotas. Sin embargo, para que se lleve a cabo de forma segura y eficaz, es necesario identificar qué pacientes pueden ser asistidos por este tipo de cuidado. Los estudios abordan la teleconsulta de enfermería en diferentes líneas de atención en la APS, como seguimiento de pacientes con COVID-19, diabetes *mellitus*, hipertensión arterial, infecciones del tracto respiratorio, infecciones de transmisión sexual, insuficiencia cardíaca, evaluación de heridas, salud mental, salud de la persona mayor y salud infantil^14^ ^)-(^ ^15^ ^),(^ ^19^ ^)-(^ ^20^ ^),(^ ^22^ ^)-(^ ^25^ ^),(^ ^27^ ^)-(^ ^30^ ^),(^ ^32^ ^)-(^ ^36^. 

Un estudio realizado en Estados Unidos demostró que la teleconsulta de enfermería, cuando se utiliza para proporcionar un tratamiento individualizado de la diabetes, tiene efectos similares en el control de la glucemia en comparación con la consulta presencial^14^. Otro estudio realizado en Reino Unido demostró que la teleconsulta telefónica de enfermería funcionó bien para revisiones de enfermedades crónicas, siendo necesaria la atención presencial solamente para evaluar aspectos físicos^25^.

Dado el potencial y difusión de la teleconsulta en la APS, se puede observar su aplicabilidad a pacientes con quejas y demandas diversas. A pesar de los diferentes enfoques, se identificó en común entre los estudios que la teleconsulta de enfermería en la APS ha sido satisfactoria, proporcionando una mejor condición clínica para el paciente. 

Factores como la falta de acceso a las TIC y/o a internet, la gravedad del cuadro clínico o la no adherencia del paciente a la consulta remota impiden su realización^22^ ^)-(^ ^24^ ^),(^ ^33^. La falta de acceso a las TIC prevalece entre la población, lo cual es una de las barreras para implementar la teleconsulta, por lo que se debe verificar previamente si el paciente cuenta con recursos tecnológicos suficientes y adecuados para realizar la teleconsulta^15^ ^),(^ ^21^ ^)-(^ ^24^ ^),(^ ^26^ ^),(^ ^33^ ^),(^ ^36^. 

El enfermero tiene prohibido realizar teleconsultas para atender situaciones de urgencia o emergencia^8^. Cuando se identifiquen señales de alerta, no se debe continuar con los cuidados, pero sí se debe aconsejar la búsqueda de un servicio de emergencia^23^ ^)-(^ ^24^ ^),(^ ^33^. Es responsabilidad del enfermero conocer la *Rede de Atenção à Saúde* (RAS) disponible en el territorio del paciente atendido, para que, en caso de necesidad de derivación, oriente el servicio adecuado para cada situación^8^.

Al ser un tipo de atención considerado reciente, es común que el paciente opte por no adherir a la teleconsulta, por lo que el formulario de consentimiento del paciente se considera un requisito para la realización de la teleconsulta^22^ ^)-(^ ^24^ ^),(^ ^36^. Según la Resolución COFEN nº 696/2022, que establece el papel de la Enfermería en la Salud Digital, estandarizando la Teleenfermería, es imprescindible el consentimiento del usuario/paciente involucrado o de su tutor legal, y que el mismo se lleve a cabo por su libre decisión, estando sujeto a la retirada en cualquier momento y, en consecuencia, a la retirada del consentimiento^6^.

### Proceso de enfermería en la teleconsulta 

La teleconsulta debe seguir el mismo método de ejecución utilizado en la consulta de Enfermería presencial, considerando el Proceso de Enfermería, incluyendo las etapas: evaluación de enfermería, diagnóstico de enfermería, planificación de enfermería, implementación y evolución de enfermería^7^ ^),(^ ^38^.

Durante la evaluación de enfermería, se recomienda que las preguntas para la recolección de datos se realicen por medio de preguntas abiertas y se recojan, preferentemente, con el paciente, solicitándose su consentimiento si es necesario incluir al familiar/cuidador. Es necesario evitar preguntas diseñadas para sí o no, para que no parezca una lista de verificación y no imposibilite la interacción entre paciente y enfermero. Además, se debe evitar el uso de preguntas que lleven al paciente a una respuesta específica, para permitir una mejor evaluación clínica^17^ ^),(^ ^21^ ^)-(^ ^22^ ^),(^ ^35^.

El hecho de que paciente y profesional no compartan un ambiente físico imposibilita el examen físico directo y limita la evaluación^19^. Pese a esto, estudios indican que por medio de la teleconsulta por video es posible inspeccionar al paciente y evaluar algunos aspectos: identificar cambios en la piel, presencia de edema, aspecto general, frecuencia respiratoria, irritación ocular, fuerza muscular y movilidad, y presencia de sangrado abundante^14^ ^),(^ ^19^ ^),(^ ^24^.

En cuanto al tipo de llamada para realizar la teleconsulta de enfermería, algunos de los estudios indicaron que la elección debe realizarse de acuerdo con la condición clínica del paciente. Se recomienda considerar algunos criterios, por ejemplo, pacientes con casos clínicamente menos complejos generalmente pueden ser atendidos por teléfono^15^ ^),(^ ^26^. La videollamada es más adecuada para pacientes con mayor complejidad y presencia de comorbilidades, así como para pacientes con discapacidad auditiva^23^ ^),(^ ^26^.

Luego de realizar la evaluación de enfermería, es necesario identificar los diagnósticos de enfermería y definir un plan de cuidados para el paciente. Cuando está capacitado, el enfermero es capaz de realizar una teleconsulta de enfermería de calidad, identificando diagnósticos precisos y problemas de enfermería, utilizando preguntas sabiamente intencionales y proponiendo intervenciones relevantes para lograr los resultados esperados^36^.

La evolución de la enfermería comprende la evaluación de los resultados alcanzados en enfermería y salud de la persona, familia, comunidad y grupos especiales. Esta etapa permite el análisis y revisión de todo el Proceso de Enfermería^38^. Los estudios demuestran que la evolución de la enfermería debe realizarse de forma detallada y clara, describiendo las etapas del proceso de enfermería^22^ ^)-(^ ^24^ ^),(^ ^27^ ^),(^ ^36^. 

Se recomienda el proceso de seguimiento reflexivo de la teleconsulta para analizar la realización de la misma, con el objetivo de mejorar las próximas consultas, y es importante identificar las principales falencias que pueden haber comprometido la calidad de la consulta, desde el análisis de los recursos tecnológicos así como aspectos relacionados con la conducta del profesional en el caso^21^.

Se observó que pocos estudios abordan el proceso de enfermería durante la teleconsulta, y que, cuando se aborda, se hace de forma disociada, es decir, mencionando solamente algunas de las etapas. Ante esto, es necesario que se desarrollen nuevos estudios enfocados a la sistematización de la asistencia de enfermería utilizando las nuevas tecnologías. También es fundamental que los profesionales estén capacitados para aplicar la sistematización de los cuidados de enfermería de forma remota.

La teleconsulta es una Práctica Avanzada de Enfermería (PAE) que exige involucramiento suficiente del profesional de enfermería para conocer al paciente como individuo, mediante la aplicación de un instrumento desarrollado y basado en un modelo teórico que utilice taxonomías estandarizadas y que sea adecuado para realizar la teleconsulta^36^.

Por lo tanto, para que la experiencia de la teleconsulta se convierta en una realidad en la APS y en el SUS, es necesaria una inversión masiva, tanto para garantizar el acceso a internet en las unidades de salud como para la obtención de computadoras y teléfonos, esenciales para el establecimiento de una comunicación satisfactoria. 

A pesar de su potencial de uso, la mayoría de las veces la teleconsulta se considera una oferta adicional, complementaria, y no es utilizada por las instituciones de salud para brindar asistencia. Por tanto, fomentar la inclusión de su uso en la práctica clínica de forma rutinaria, especialmente en la APS, y en la formación de los futuros profesionales de la salud, es fundamental para lograr un uso adecuado.

Esta revisión destacó la evidencia científica de varios países cuya APS está organizada de diferentes maneras, según aspectos económicos, sociales y políticos y con base en la comprensión local del proceso salud-enfermedad. En el contexto europeo, el concepto de atención primaria puede entenderse como un nivel de atención entre cuidado informal y cuidado hospitalario. Por otro lado, en algunos países americanos, la APS se refiere a un conjunto específico de actividades de servicios de salud dirigidos a la población pobre. En los países asiáticos, la reformulación de la APS se ha venido produciendo más recientemente, apuntando a un sistema accesible y equitativo. 

Los aportes de este estudio al área de Enfermería y salud se basan en el impacto de la teleconsulta de enfermería en los últimos años, principalmente debido a la pandemia. Estudios como este son incentivos para reflexionar sobre los nuevos escenarios de cuidado en los que debe actuar el enfermero, además de incentivar la implementación de la teleconsulta en la APS.

Como limitación, podemos mencionar el análisis y la síntesis de datos realizados únicamente de forma descriptiva. Combinar datos de diferentes tipos de estudios es un proceso desafiante que puede generar sesgos en la preparación de los resultados de la revisión. A pesar de las limitaciones, esta revisión destaca el rigor metodológico requerido por el JBI y el mapeo de evidencia sobre la teleconsulta de enfermería. 

## Conclusión

La teleconsulta de enfermería en atención primaria es una forma emergente de brindar atención médica. Cuando se resuelven las dificultades técnicas y organizativas, se puede ahorrar tiempo y recursos a pacientes y profesionales, alcanzando niveles satisfactorios de resolución de problemas, sin necesidad de desplazarse a Unidades Básicas de Salud. Sin embargo, para su implementación es necesario capacitar al enfermero en las siguientes habilidades: comunicación, clínica, tecnológica, ética y aquellas relacionadas con la infraestructura del ambiente de teleconsulta. 

Se identificó la necesidad de competencias profesionales para afrontar la teleconsulta de enfermería, lo que exige un proceso de formación continua que permee los espacios de la formación académica, hasta la práctica profesional del enfermero.
